# Targeting the NANOG/HDAC1 axis reverses resistance to PD-1 blockade by reinvigorating the antitumor immunity cycle

**DOI:** 10.1172/JCI147908

**Published:** 2022-03-15

**Authors:** Se Jin Oh, Hyo-Jung Lee, Kwon-Ho Song, Suyeon Kim, Eunho Cho, Jaeyoon Lee, Marcus W. Bosenberg, Tae Woo Kim

**Affiliations:** 1 BK21 Graduate Program, Department of Biomedical Sciences and; 2 Department of Biochemistry and Molecular Biology, Korea University College of Medicine, Seoul, South Korea.; 3 Department of Cell Biology, Daegu Catholic University School of Medicine, Daegu, South Korea.; 4 College of Science, College of Social Sciences and Humanities, Northeastern University, Boston, Massachusetts, USA.; 5 Department of Pathology and; 6 Department of Dermatology, Yale University School of Medicine, New Haven, Connecticut, USA.; 7 NEX-I Inc., Seoul, South Korea.

**Keywords:** Oncology, Therapeutics, Cancer immunotherapy

## Abstract

Immune checkpoint blockade (ICB) therapy has shifted the paradigm for cancer treatment. However, the majority of patients lack effective responses because of the emergence of immune-refractory tumors that disrupt the amplification of antitumor immunity. Therefore, the identification of clinically available targets that restrict antitumor immunity is required to develop potential combination therapies. Here, using transcriptomic data on patients with cancer treated with programmed cell death protein 1 (PD-1) therapy and newly established mouse preclinical anti–PD-1 therapy–refractory models, we identified NANOG as a factor restricting the amplification of the antitumor immunity cycle, thereby contributing to the immune-refractory feature of the tumor microenvironment (TME). Mechanistically, NANOG induced insufficient T cell infiltration and resistance to CTL-mediated killing via the histone deacetylase 1–dependent (HDAC1-dependent) regulation of CXCL10 and MCL1, respectively. Importantly, HDAC1 inhibition using an actionable agent sensitized NANOG^hi^ immune-refractory tumors to PD-1 blockade by reinvigorating the antitumor immunity cycle. Thus, our findings implicate the NANOG/HDAC1 axis as a central molecular target for controlling immune-refractory tumors and provide a rationale for combining HDAC inhibitors to reverse the refractoriness of tumors to ICB therapy.

## Introduction

Immune checkpoint blockade (ICB) therapy elicits a marked clinical response in patients with various tumor types, changing the paradigm for cancer treatment (1–3). However, despite the developing field’s potential to revolutionize cancer treatment, the emergence of immune-refractory tumors has limited its clinical success (4, 5). Among the diverse causes of the development of immune-refractory tumors, the cancer immunoediting theory has attracted attention, as it can explain the emergence of tumors refractory to antitumor immunity (6). Indeed, previous studies have provided evidence that cancer immunoediting triggered the adaptation of tumor cells to the host’s immune system, thereby contributing to the generation of cancer cells with better survival advantages (7, 8). In this regard, we had found that immune selection by immunotherapy, such as vaccination or adoptive T cell transfer (ACT), drives the evolution of tumors toward immune-refractory states, such as resistance of tumor cells to T cell–mediated killing (9–12). Interestingly, the immune-refractory tumors were resistant to T cell–mediated killing and restricted host antitumor immunity (13). Thus, understanding the molecular mechanism that disrupts antitumor immunity could reveal potential targets for overcoming clinical limitations to ICB therapy.

Theoretically, the immune system should be capable of eradicating tumor cells through an acquired immune response executed by tumor-reactive CD8^+^ T cells. A series of stepwise events, called the antitumor immunity cycle, is required for tumor cell clearance by the immune system (14). Notably, the dysfunction of single or multiple steps in the antitumor immunity cycle is observed in many patients who do not respond to ICB therapy (14, 15), suggesting that the blockade of any steps of the antitumor immunity cycle could be a crucial obstacle, resulting in resistance to ICB therapy. Thus, it is important to identify the cause of blockade of the antitumor immunity cycle to overcome the resistance to ICB therapy.

Accumulating evidence has indicated that the oncogenic pathways of tumors not only promote tumorigenesis but also interfere with the processes essential for effective antitumor immunity, such as T cell trafficking to tumors and T cell–mediated killing of tumor cells (16–19). For example, hyperactivation of AKT signaling by phosphatase and tensin homolog (PTEN) loss impedes the trafficking of effector T cells to tumors, reduces the sensitivity of melanoma cells to T cell–mediated killing, and is correlated with inferior outcomes for patients treated with ICB (19, 20). In addition, several studies have revealed that tumors commonly hijack various epigenetic mechanisms to escape immune restriction (21). Among these epigenetic regulators, histone deacetylases (HDACs) have been found to regulate a variety of effects in the T cell–mediated antitumor response. For example, dysregulated HDACs in tumors not only decreased T cell trafficking to tumors but inhibited cell death in response to T cell–mediated killing (22). Notably, HDAC inhibition using pharmacological agents increased the levels of T cell chemoattractants and tumor infiltration into multiple lung adenocarcinomas, with a correlated sensitization to anti–programmed cell death protein 1 (anti–PD-1) therapy. Building on these preclinical studies, HDAC inhibition in combination with ICB therapy is currently being explored in multiple clinical trials (23). For instance, promising results have been obtained by combining an HDAC1 inhibitor and anti–PD-1 to treat patients with metastatic uveal melanoma (NCT02697630; https://clinicaltrials.gov/ct2/show/NCT02697630) or advanced/metastatic non–small cell lung cancer (NCT02638090; ref. 24). These results indicate that targeting oncogenic pathways that restrict the antitumor immunity cycle could be a potential strategy to overcome resistance to ICB therapy. Thus, discovering an oncogenic pathway that can be targeted by clinically available drugs is needed to develop therapeutic strategies to overcome the clinical limitations of ICB therapy.

The pluripotency transcription factor NANOG, known as a key regulator of embryonic development and cellular reprogramming, has been reported to be broadly expressed in human cancers (25). Functional studies have provided strong evidence that NANOG plays a vital role in malignant disease, correlating with various malevolent properties such as tumorigenicity, self-renewal, invasiveness, and multimodal therapeutic resistance (26, 27). Furthermore, we identified NANOG as a key intrinsic factor that could induce resistance to CTL-mediated immunotherapy and found that NANOG-mediated therapeutic resistance is dependent on HDAC1 (28). Notably, inhibition of the NANOG/HDAC1 axis reversed the resistance to CTL-based immunotherapy in tumor cells and led to long-term control of the disease (28). These findings suggest that the strategies impeding the NANOG/HDAC1 axis may help conquer the clinical limitations of ICB therapy. However, despite the relevance of NANOG expression in ICB therapy–refractory cancer, the precise mechanism by which NANOG could trigger resistance to ICB therapy, especially through HDAC1-mediated epigenetic reprogramming, is not well understood.

Here, we integrated analyses of patients with cancer from The Cancer Genome Atlas (TCGA) cohort and newly established mouse preclinical anti–PD-1 therapy–refractory models (CT26 P3 or YUMM2.1 P3) and found that elevated NANOG expression in tumors could reprogram the tumor microenvironment (TME) into one that was immunologically nonresponsive to tumors. Our data indicate that NANOG induction in tumor cells switched the immune phenotypes in the TME from an immune-stimulatory to an immune-refractory feature by blocking the antitumor immunity cycle via restriction of T cell trafficking to tumors and by cytotoxic T lymphocyte–mediated (CTL-mediated) killing of tumor cells, thereby driving resistance to PD-1 blockade. Importantly, the phenotype of these cells is critically dependent on HDAC1, the main intermediator of NANOG-mediated regulation of the expression of multiple genes. Thus, we provide proof-of-concept evidence that HDAC1 inhibition by pharmacological interventions could suppress tumor progression and sensitize NANOG^hi^-refractory tumors to anti–PD-1 therapy.

## Results

### NANOG is associated with an immune-refractory feature of the TME in patients with cancer.

**Figure 1:**
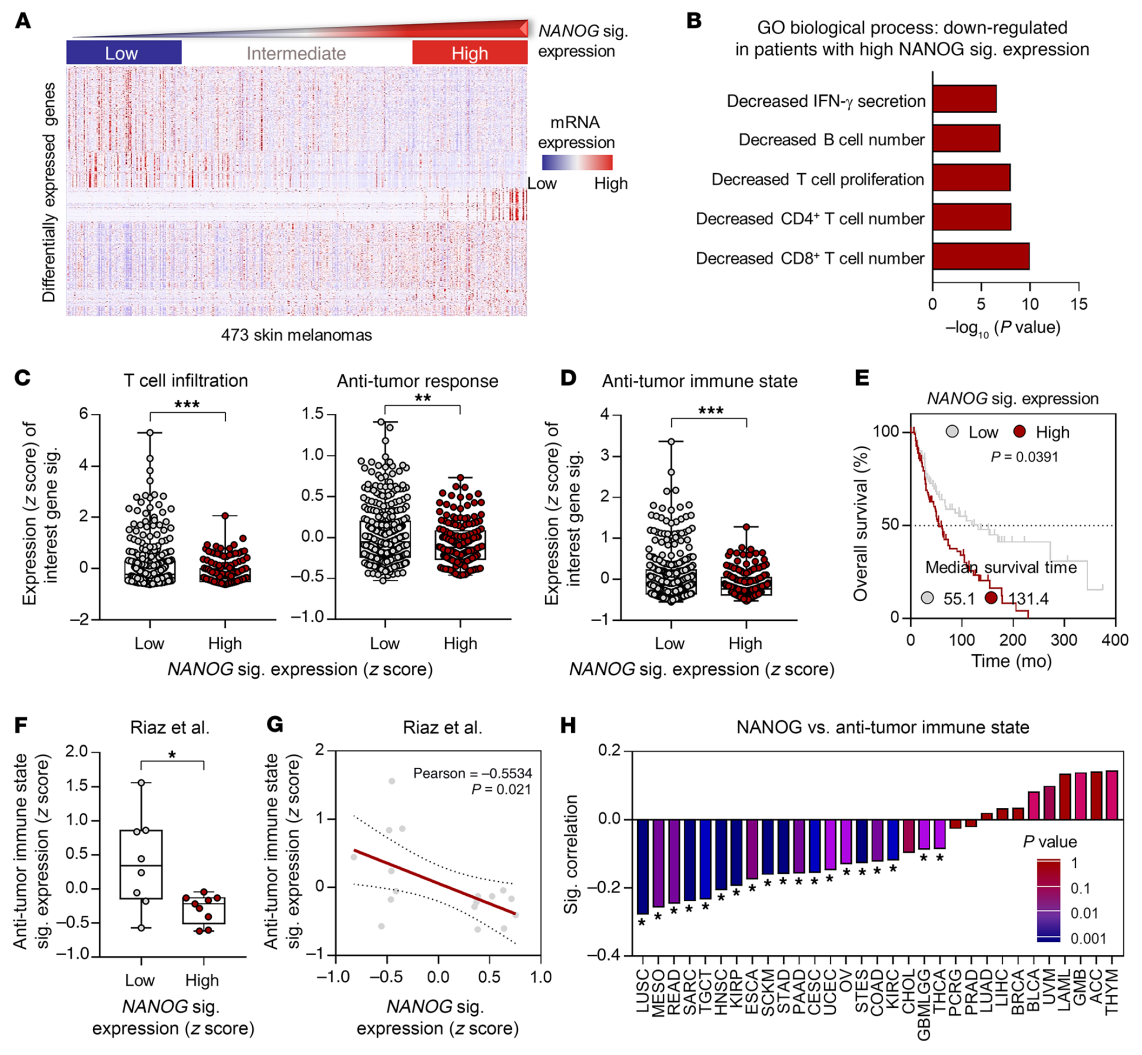
NANOG is inversely associated with the antitumor immune state of the TME in patients with cancer. (**A**) Expression of the top 600 differentially expressed genes in patients with NANOG^hi^ or NANOG^lo^ melanoma. (**B**) GO term enrichment analysis for the top 5 biological processes controlled by differentially expressed genes among patients with high NANOG signature expression. (**C** and **D**) Comparisons of expression levels of T cell infiltration, antitumor response, and antitumor immune state signatures in NANOG^lo^ (*n =* 315) and NANOG^hi^ (*n =* 158) patients. (**E**) Kaplan-Meier analysis of overall survival (calculated as months to death or months to last follow-up) and median cutoffs values for NANOG signature expression levels (NANOG^hi^ > median; NANOG^lo^ < median, *P =* 0.0391). (**F**) Comparison of expression levels of antitumor immune state signatures in nonresponders with low levels (low, *n =* 8) or high levels (high, *n =* 9) of NANOG signature expression. The 25th and 75th percentiles were used as cutoff thresholds. (**G**) Pearson’s correlation between NANOG signature expression levels and antitumor immune states in nonresponders. (**H**) Correlation plot of NANOG and antitumor immune state signatures in pan-tumor types. Correlation and 2-tailed *P* values were assessed using the Pearson’s correlation coefficient and the unpaired Student’s *t* test. In the box plots, the top and bottom edges of boxes indicate the first and third quartiles, respectively; the center lines indicate the medians; and the ends of the whiskers indicate the maximum and minimum values, respectively. *P < 0.05, **P < 0.01, and ***P < 0.001, by unpaired, 2-tailed Student’s *t* test (**C**, **D**, and **F**). sig., signature.

Previously, we defined a *NANOG* signature to acquire a more reliable readout indicating NANOG expression in tumor cells (13). To understand the underlying mechanism of NANOG-driven resistance to PD-1 blockade, we first assessed the transcriptomes of TCGA melanoma patients with varying *NANOG* signature expression. We analyzed the top 600 differentially expressed genes in patients who were *NANOG*^hi^ versus those who were *NANOG*^lo^ (adjusted *P* < 0.001; Supplemental Table 1; supplemental material available online with this article; https://doi.org/10.1172/JCI147908DS1) and found that the *NANOG* signature was inversely associated with genes controlling IFN-γ secretion, T cell proliferation, and infiltration in the TME (Figure 1, A and B). High or low T cell antitumor immune responses could be predicted by determining the expression signature scores of 7 gene sets that have been reported to be indicators of increased T cell infiltration and the antitumor response (hereafter referred to as antitumor immune states; refs. 29–32). Interestingly, we found that the *NANOG* signature was inversely associated with the gene signature that represents T cell infiltration and the antitumor response (Figure 1, C and D). These results indicate that elevated NANOG expression in melanoma cells could shape the TME into expressing an immune-refractory feature, such as insufficient T cell trafficking to tumors and resistance of tumor cells to T cell–mediated killing. We next examined the clinical relevance of NANOG in TCGA patients and found that Kaplan-Meier plots indicated that patients with high *NANOG* signature levels had significantly worse overall survival rates (Figure 1E). Together, our results suggest that elevated NANOG expression in tumors could result in restrained antitumor immunity in the TME as well as poor clinical outcomes.

We previously reported that NANOG could contribute to a poor response to anti–PD-1 therapy (13). On the basis of our observations, we further questioned whether NANOG confers a poor response to anti–PD-1 therapy by inducing immune-refractory features in the TME. By analyzing *NANOG* and the antitumor immune state signature in transcriptomic data on patients with melanoma classified as responders or nonresponders to anti–PD-1 treatment (33), we found that the antitumor immune state signature scores were significantly lower in nonresponders than in responders (Supplemental Figure 1). We also found a strong negative correlation between the *NANOG* signature and antitumor immune state expression in the nonresponders (Figure 1, F and G). Furthermore, the above results were reproduced in 2 independent data sets of patients with melanoma treated with anti–PD-1, including the Gide et al. (34) and Liu et al. (35) cohorts (Supplemental Figure 2). Notably, the NANOG signature was inversely associated with the antitumor immune state signature in multiple tumor types, such as lung squamous cell carcinoma (LUSC), mesothelioma (MESO), rectum adenocarcinoma (READ), sarcoma (SARC), testicular germ cell tumors (TGCTs), head and neck squamous cell carcinoma (HNSC), kidney renal papillary cell carcinoma (KIRP), esophageal carcinoma (ESCA), skin cutaneous melanoma (SKCM), stomach adenocarcinoma (STAD), pancreatic adenocarcinoma (PAAD), cervical and endocervical cancers (CESCs), uterine corpus endometrial carcinoma (UCEC), ovarian serous cystadenocarcinoma (OV), stomach and esophageal carcinoma (STES), colon adenocarcinoma (COAD), kidney renal clear cell carcinoma (KIRC), glioma (GMBLGG), and thyroid carcinoma (THCA) (Figure 1H). Taken together, our results indicate that NANOG is associated with an immune-refractory feature of the TME, including non–T cell–inflamed tumors and resistance to CTL-mediated killing, suggesting that NANOG may drive resistance to ICB therapy by inducing an immune-refractory feature in the TME.

### An ICB therapy–refractory tumor model displays an immune-refractory feature in the TME.

**Figure 2:**
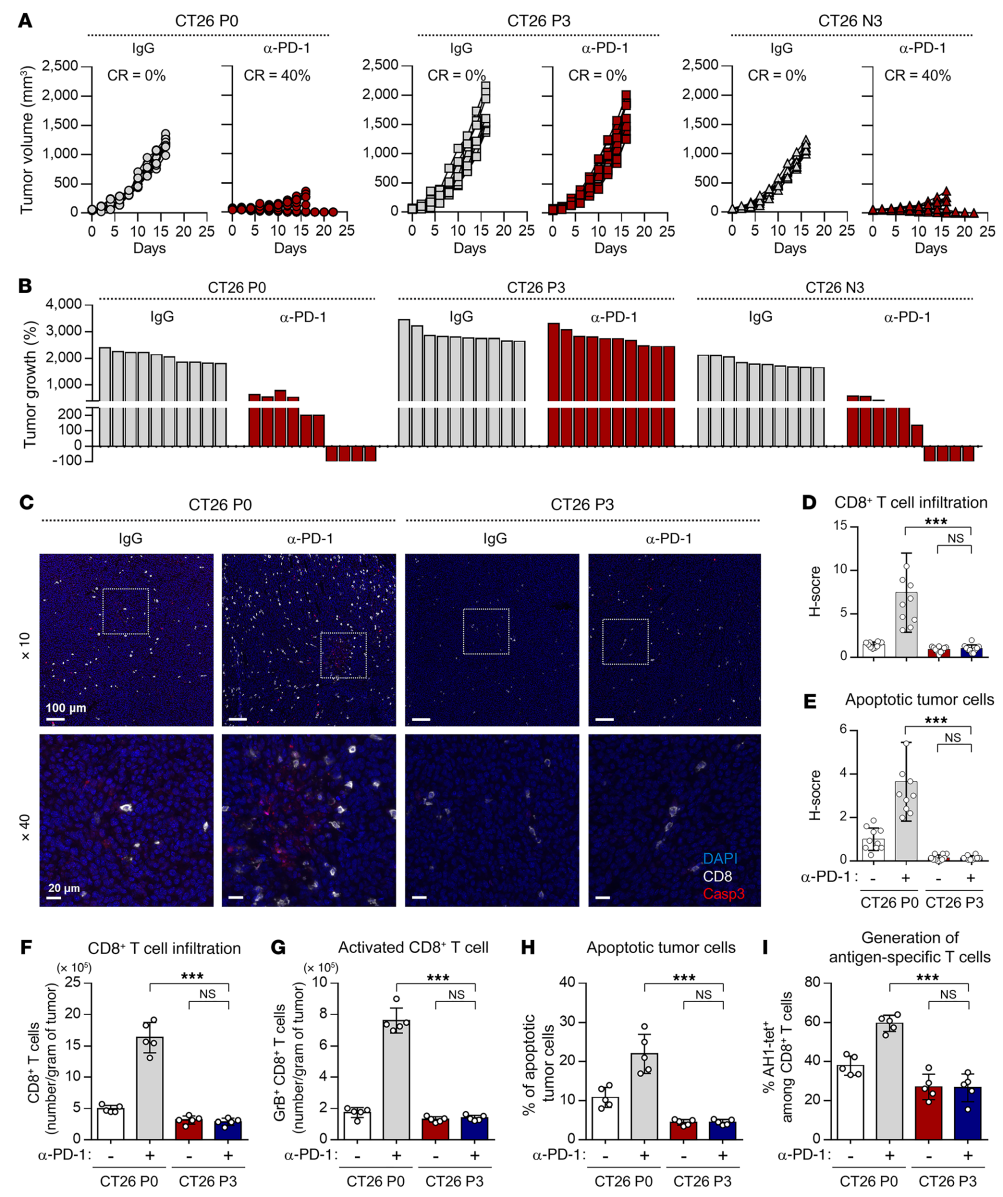
CT26 P3 cells display the immune-refractory feature of the TME. (**A**–**I**) CT26 P0, P3, or N3 tumor–bearing mice were treated with IgG or anti–PD-1 (α–PD-1) antibody. (**A**) Tumor growth curves and (**B**) changes in tumor volume 17 days after challenge compared with baseline. CR, complete response. (**C**) Formalin-fixed, paraffin-embedded (FFPE) sections of CT26 P0 or P3 tumors treated with IgG or anti–PD-1 antibody were stained with the indicated markers by pseudo-coloring. The indicated markers are shown on the right. Scale bars: 100 μm and 20 μm (enlarged insets). (**D**) Frequency of tumor-infiltrating CD8^+^ T cells. (**E**) Frequency of apoptotic cells in the tumors. (**F**) Flow cytometric profiles of the tumor-infiltrating CD3^+^CD8^+^ T cells. (**G**) Ratio of granzyme B^+^ to tumor-infiltrating CD3^+^CD8^+^ T cells. (**H**) Frequency of apoptotic cells in the tumors. (**I**) Quantification of antigen-specific CTLs in spleens from the tumor-bearing mice. Ten mice from each group were used for in vivo experiments. Results shown in the graphs represent 3 independent experiments performed in triplicate. (**D**–**I**) Data represent the mean ± SD. ***P < 0.001, by 1-way ANOVA.

To understand the underlying mechanisms responsible for the NANOG-driven immune-refractory feature of the TME in ICB therapy, we conducted 3 rounds of in vivo selection following PD-1 blockade to establish new anti–PD-1 therapy–refractory tumor models from a CT26 cell line (designated as CT26 P0 cells) or a YUMM2.1 cell line (designated as YUMM2.1 P0 cells), both of which have frequently been used as preclinical tumor models for the study of cancer biology and tumor immunology as well as evaluating the efficacy of ICB therapy (Supplemental Figure 3 and refs. 36–39). These cells treated with anti–PD-1 were termed P3, whereas those treated with IgG as a negative control were termed N3. To validate our model, we treated tumor-bearing mice with anti–PD-1 (Supplemental Figure 4). Anti–PD-1 treatment showed a remarkable therapeutic effect in both CT26 P0 and CT26 N3 tumor–bearing mice (Figure 2, A and B). In contrast, in CT26 P3 tumor–bearing mice, anti–PD-1 treatment had no effect on tumor growth (Figure 2, A and B). Consistently, YUMM2.1 P3 cells displayed resistance to anti–PD-1 treatment (Supplemental Figure 5A). Thus, our data indicate that the resistance to anti–PD-1 therapy seen in patients was conserved in our ICB therapy–refractory tumor models.

The response to ICB therapy in patients is determined by immune features in the TME that are regulated by antitumor immunity, such as T cell trafficking to tumors and T cell–mediated killing of tumor cells (known as the antitumor immunity cycle; ref. 14). To investigate whether immune-refractory tumors displayed an immune-refractory feature in the TME, we performed multiplex IHC (mIHC) with computational image-processing workflows. Compared with CT26 P0 tumors, the CT26 P3 tumors had decreased CD8^+^ T cell infiltration and apoptotic tumor cell death under anti–PD-1 treatment (Figure 2, C–E). Consistently, flow cytometric analysis revealed that the overall numbers of CD8^+^ T cells and tumor-reactive CD8^+^ T cells expressing granzyme B and the percentage of apoptotic tumor cells were significantly lower in the CT26 P3 cells than in the CT26 P0 cells (Figure 2, F–H). Co-linearly, YUMM2.1 P3 cells showed a decrease in the overall number of overall CD8^+^ T cells and tumor-reactive CD8^+^ T cells and an increase in the percentage of apoptotic tumor cells compared with YUMM2.1 P0 cells (Supplemental Figure 5, B–D). These data clearly demonstrate that P3 tumors displayed the immune-refractory feature of the TME, which is the key cause of resistance to anti–PD-1 therapy in clinical settings.

Some of the mechanisms controlling TME resistance to anti–PD-1 therapy include the downregulation or loss of expression of MHC class I molecules, which could account for the decreased CTL recognition of tumor cells (40). Therefore, we assessed MHC class I (H2-K^d^, D^d^) expression and found that its expression in CT26 P0 and P3 cells was virtually identical, regardless of immune selection of tumor cells (Supplemental Figure 6A). We next tested the possibility that immune selection imposed by anti–PD-1 therapy may alter the function of antigen-specific CTLs. We found that there was no difference between P0 and P3 cells in terms of CTL effector cytokine (IFN-γ) production of AH1-specific CTLs mixed with AH1-epitopic peptide–loaded tumor cells, suggesting that P0 and P3 cells had similar T cell activation capabilities and potentials to be recognized by the CTLs (Supplemental Figure 6B). Instead, CT26 P3 cells appeared more resistant to lysis by the CTLs or granzyme B than did CT26 P0 cells (Supplemental Figure 7, A and B). Therefore, our data suggest that anti–PD-1–mediated immune selection facilitated the enrichment of a subset of tumor cells with increased resistance to CTL killing rather than CTL recognition or activation.

Accumulating evidence suggests that tumor cell death in cancer treatment can lead to the release of tumor antigens that subsequently prime the auto-loop of the antitumor immune response (41–44). We next studied the generation of antigen-specific T cells in CT26 P0 and P3 cells. We isolated cells from the spleens of mice that received anti–PD-1 therapy and assessed tumor antigen–specific (AH1^+^-specific) CD8^+^ T cells. Notably, tumor antigen–specific CD8^+^ T cells were decreased in CT26 P3–bearing mice compared with CT26 P0–bearing mice (Figure 2I), suggesting that decreased generation of antigen-specific T cells could be due to the resistance of CT26 P3 cells to CTL-mediated killing. Taken together, our results indicate that CT26 P3 cells displayed an immune-refractory feature of the TME observed in clinical practice by restricting self-amplifying antitumor immunity to anti–PD-1 therapy.

### NANOG expression in tumor cells determines the response to anti–PD-1 therapy by altering the immune feature of the TME.

**Figure 3:**
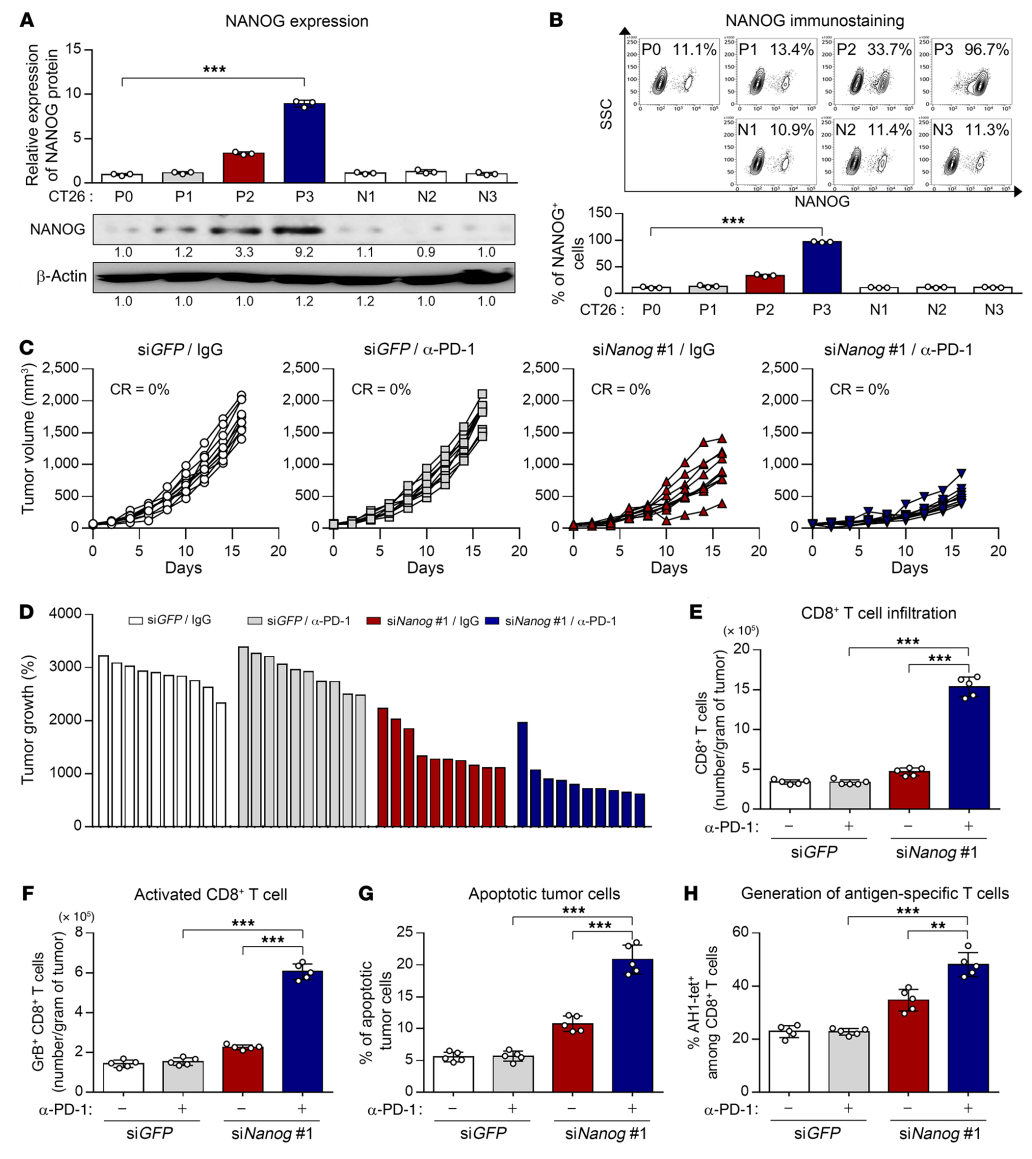
NANOG repression enhances the response to anti–PD-1 therapy by inducing the immune-stimulatory feature in the TME. (**A**) Top: Quantification of NANOG expression in tumor cells at different stages of immune selection (P0–P3). Parallel stages without selection are labeled as N1–N3. Bottom: Representative Western blots. (**B**) Flow cytometric analysis of NANOG^+^ tumor cells and quantification of the frequency of NANOG^+^ tumor cells. (**C**–**H**) CT26 P3 tumor–bearing mice were administered si*GFP* or si*Nanog* with or without anti–PD-1 antibody treatment. (**C**) Tumor growth curves and (**D**) changes in tumor volume 17 days after challenge compared with baseline. (**E**) Flow cytometric profiles of tumor-infiltrating CD3^+^CD8^+^ T cells. (**F**) Ratio of granzyme B^+^ to tumor-infiltrating CD3^+^CD8^+^ T cells. (**G**) Frequency of apoptotic cells in the tumors. (**H**) Quantification of antigen-specific CTLs in spleens from tumor-bearing mice. Ten mice from each group were used for in vivo experiments. Results in the graphs represent 3 independent experiments performed in triplicate. Data represent the mean ± SD. **P < 0.01 and ***P < 0.001, by 2-tailed Student’s *t* test (**A** and **B**) and 1-way ANOVA (**E**–**H**).

On the basis of our observations, we reasoned that differences in immune features of the TME may be due to their dissimilarities in the oncogenic pathway mediated by an intrinsic factor of tumor cells. We previously reported that NANOG, a crucial intrinsic factor, may be a potential target to overcome the resistance to anti–PD-1 therapy (13). To characterize the role of NANOG in immune-refractory features of the TME in an ICB therapy–refractory tumor model, we first assessed the expression of NANOG in CT26 cells at different rounds of immune selection (P0 to P3) and found a stepwise increase in NANOG expression from P0 to P3 (Figure 3A). The overall increase in NANOG expression in the CT26 P3 cell line was likely due to enrichment of NANOG^+^ cells, as opposed to upregulation of NANOG expression itself, as the frequency of NANOG^+^ cells rose from approximately 11% in the CT26 P0 cell line to approximately 96% in the CT26 P3 cell line (Figure 3B). In contrast, we detected no significant increase in NANOG levels in tumor cells under selection with IgG (N1–N3; Figure 3, A and B). Consistently, NANOG expression was increased in another ICB therapy–refractory tumor model (YUMM2.1 P3 cells) (Supplemental Figure 8). Thus, our data indicate that anti–PD-1–mediated immune selection depletes tumor cells lacking NANOG while enriching tumor cells that express NANOG, suggesting that NANOG expression in tumor cells could confer a survival advantage under the immune selection pressure imposed by anti–PD-1 therapy.

To further investigate whether NANOG expression in tumor cells modulates the immune feature of the TME, we treated CT26 P3 tumor–bearing mice with intravenously administered chitosan nanoparticles carrying a *Nanog*- or *GFP*-targeting siRNA along with anti–PD-1 (Supplemental Figures 9 and 10). We found that IgG- and anti–PD-1–treated CT26 P3 tumors displayed similar growth rates under control si*GFP* treatment. However, si*Nanog* treatment suppressed the growth of IgG- and anti–PD-1–treated CT26 P3 tumors. Of note, combined treatment with anti–PD-1 and si*Nanog* drastically retarded tumor growth (Figure 3, C and D). Notably, we found that knockdown of NANOG significantly promoted the overall number of tumor-infiltrating CD8^+^ T cells (TILs) or tumor-reactive CD 8^+^ TILs in tumors treated with anti–PD-1 (Figure 3, E and F). Many reports suggested that elevated infiltration of tumor-reactive CD8^+^ T cells could lead to tumor cell death that subsequently primes the auto-loop of the antitumor immune response (14). Consistently, we found that the percentage of apoptotic tumor cells was increased by combined treatment compared with either single treatment alone (Figure 3G). Indeed, the generation of tumor antigen–specific CD8^+^ T cells was elevated in the combination treatment group compared with the other treatment groups (Figure 3H). Conversely, the *Nanog* gene–transduced CT26 P0 cells phenocopied the CT26 P3 cells, displaying an immune-refractory feature in the TME (Supplemental Figure 11, A–H). Together, these results indicate that NANOG expression in tumor cells could switch the immune phenotype of the TME from immune-stimulatory to immune-refractory feature by controlling both T cell infiltration into tumors and CTL-mediated killing of tumor cells. Therefore, therapeutic strategies targeting NANOG could reverse the immune-refractory feature of the TME, thereby improving the efficacy of ICB therapy.

### NANOG blocks CD8^+^ T cell infiltration into tumors through HDAC1-mediated epigenetic repression of CXCL10 expression.

**Figure 4:**
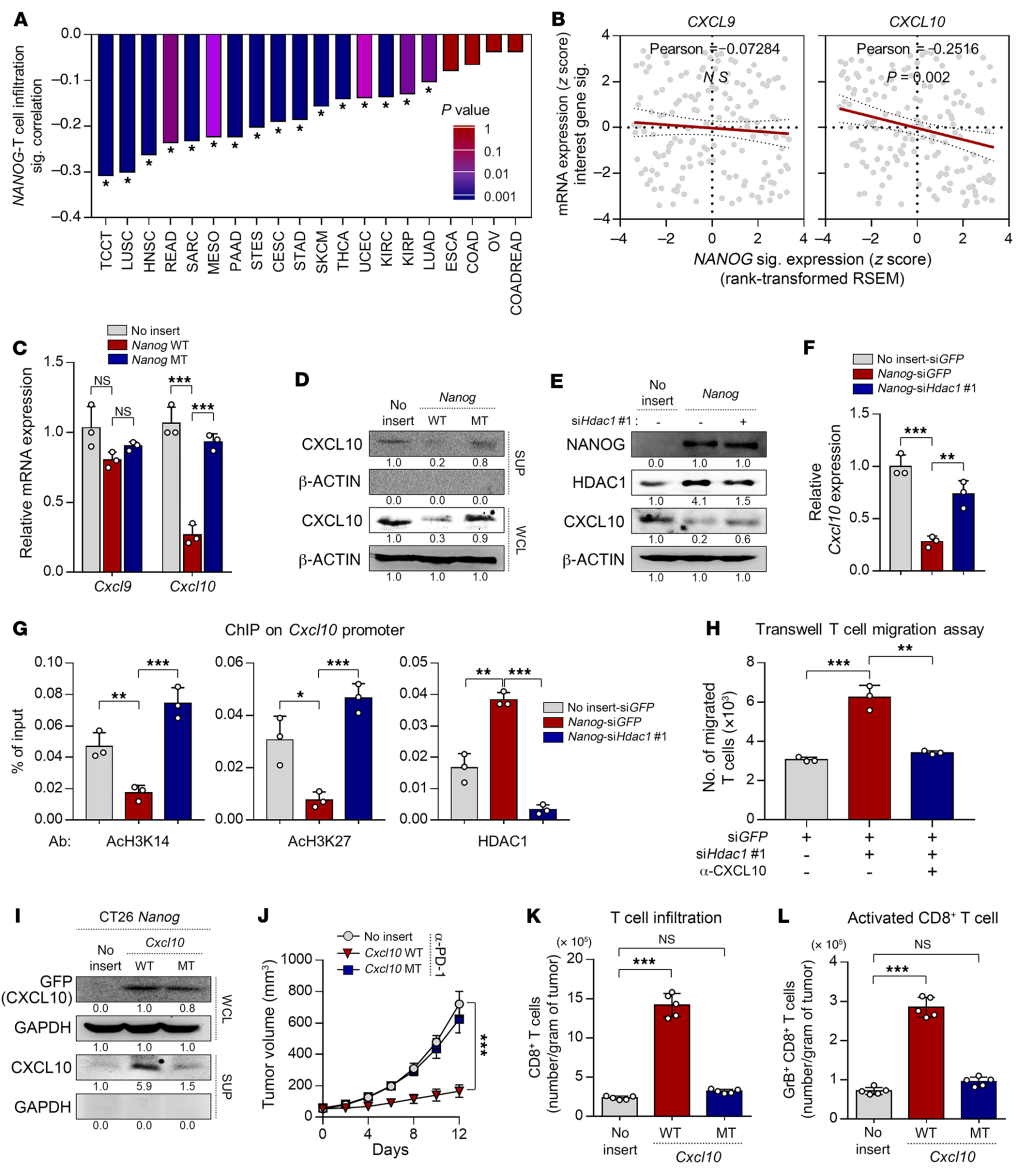
NANOG blocks CD8^+^ T cell infiltration through HDAC1-mediated epigenetic repression of CXCL10. (**A**) Correlation plot of NANOG and T cell infiltration signatures in pan-tumor types. Correlation and 2-tailed *P* values were assessed using the Pearson’s correlation coefficient and unpaired Student’s *t* test. (**B**) Pearson’s correlation of NANOG signature expression with indicated transcripts of T cell–recruiting chemokines. RSEM, relative SEM. (**C**) qPCR analysis of *Cxcl9* and *Cxcl10* mRNA expression. (**D**) Western blot analysis of the expression of CXCL10. (**E**–**G**) CT26-no insert or CT26-*Nanog* cells were transfected with si*GFP* or si*Hdac1*. WCL, whole-cell lysate; SUP, supernatant. (**E**) Western blot analysis of CXCL10 expression. (**F**) qPCR analysis of *Cxcl10* mRNA expression. (**G**) Relative occupancy of AcH3K14, AcH3K27, and HDAC1 in the *Cxcl10* promoters was assessed by qChIP analysis. The ChIP data values represent ratios relative to the input. (**H**) Transwell T cell migration assay using CT26-*Nanog*-si*GFP* or CT26-*Nanog*-si*Hdac1* cells were treated with IgG or anti-CXCL10. (**I**) Western blot analysis of the expression of GFP (CXCL10) in CT26-no insert or CT26-*Nanog* WT cells transduced with *Cxcl10* WT or *Cxcl10* MT. (**J**–**L**) CT26-no insert, CT26-*Nanog*-*Cxcl10* WT, or CT26-*Nanog*-*Cxcl10* MT tumor–bearing mice were treated with anti–PD-1 antibody. (**J**) Tumor growth curves. (**K**) Flow cytometric profiles of tumor-infiltrating CD3^+^CD8^+^ T cells. (**L**) Ratio of granzyme B^+^ to tumor-infiltrating CD3^+^CD8^+^ T cells. Five mice from each group were used for in vivo experiments. (**D**, **E**, and **I**) β-Actin was used as an internal loading control. Results in the graphs represent 3 independent experiments performed in triplicate. Data represent the mean ± SD. *P < 0.05, **P <0.01, and ***P < 0.001, by 1-way ANOVA (**C**, **F**, **G**, **H**, **K**, and **L**) and 2-way ANOVA (**J**).

We next attempted to elucidate the underlying mechanism responsible for the NANOG-driven immune-refractory feature of the TME. Previous studies indicated that CD8^+^ T cell infiltration into tumors was required to initiate the subsequent auto-loop of antitumor immunity and was also regulated by special chemokines, such as CXCL9 and CXCL10 (45–48). Interestingly, we found that the *NANOG* signature was inversely associated with the T cell infiltration signature in multiple tumor types (Figure 4A). Among T cell recruitment–related chemokine genes, we found that the *NANOG* signature was inversely associated with transcript expression of *CXCL10*, but not of other chemokines (Figure 4B). These results suggest that NANOG could impair T cell recruitment to tumors by repressing the T cell–recruiting chemokine CXCL10 in the TME.

As NANOG regulates multiple gene expression programs, we questioned whether NANOG regulates CXCL10 expression through its transcriptional function. To assess this, we used a mutant form of *Nanog* (*Nanog* MT) that was previously found to have weak transcriptional activity (10). When we transfected CT26 P0 cells with wild-type *Nanog* (*Nanog* WT), CXCL10 mRNA and protein expression levels were reduced, whereas the transfection of *Nanog* MT had no significant impact on CXCL10 expression (Figure 4, C and D), indicating that NANOG-mediated CXCL10 regulation was dependent on the transcriptional activity of NANOG. Previously, it was demonstrated that HDAC1 is a key element in NANOG-mediated transcriptional repression (28), and HDAC-mediated histone deacetylation is involved in the increase in CXCL10 expression (49, 50). Hence, we asked whether HDAC1 is a crucial intermediator in CXCL10 downregulation mediated by NANOG. To address this question, we measured the levels of CXCL10 protein and mRNA in CT26-no insert-si*GFP*, CT26-*Nanog-*si*GFP*, and CT26-*Nanog-*si*Hdac1* cells (Supplemental Figure 12). We found that CXCL10 protein and mRNA transcript levels were significantly decreased and that these decreased levels of CXCL10 mediated by NANOG were reversed upon silencing of HDAC1 (Figure 4, E and F). Because NANOG caused the transcriptional repression of CXCL10 in an HDAC1-dependent manner, we reasoned that decreased expression of CXCL10 may have been due to HDAC1-mediated epigenetic silencing. We previously demonstrated that NANOG caused a decrease in AcH3K14 and AcH3K27 through transcriptional activation of HDAC1 (28). Notably, ChIP quantitative PCR (qPCR) showed that NANOG expression caused loss of AcH3K14 and AcH3K27 occupancy on the promoter region of *Cxcl10*, and these histone modification events were reversed by HDAC1 knockdown (Figure 4G). Consistent with these results, the present study showed that HDAC1 was more enriched in the *Cxcl10* promoters in CT26-*Nanog-*si*GFP* cells compared with CT26-no insert-si*GFP* cells (Figure 4G). These results demonstrate that NANOG could downregulate expression of the *CXCL10* gene via HDAC1-mediated epigenetic silencing.

To assess the potential role of CXCL10 in the NANOG-mediated non–T cell–inflamed immune phenotype in the TME, we performed antibody-mediated neutralization of CXCL10 in CT26-*Nanog-*si*GFP* cells and CT26-*Nanog-*si*Hdac1* cells. We found that elevated T cell infiltration mediated by HDAC1 knockdown was completely reversed upon neutralization of CXCL10 (Figure 4H), suggesting that the NANOG/HDAC1 axis induced the non–T cell–inflamed immune phenotype in the TME in a CXCL10-dependent manner. We next questioned whether elevated T cell trafficking could overcome NANOG-driven resistance to ICB therapy. To estimate this, we used a WT *Cxcl10* (*Cxcl10* WT) or a mutant form of *Cxcl10* (*Cxcl10* MT) that was previously found to have nonsecretory properties (Figure 4I and ref. 51). Whereas the addition of *Cxcl10* WT sensitized CT26-*Nanog* tumors to PD-1 blockade and accelerated overall CD8^+^ T cell or tumor-reactive CD8^+^ cell recruitment, *Cxcl10* MT had no impact on these parameters (Figure 4, J–L). Although we observed significant suppression of tumor growth in *NANOG-Cxcl10* WT tumor–bearing mice compared with mice in the other groups, these tumors continued to grow (Figure 4J). Therefore, these results indicate that NANOG induces the immune-refractory feature in the TME by controlling not only T cell infiltration but also other steps of the antitumor immunity cycle

### Resistance of tumor cells to T cell–mediated killing by the NANOG/HDAC1 axis is essential for inducing the immune-refractory feature in the TME.

**Figure 5:**
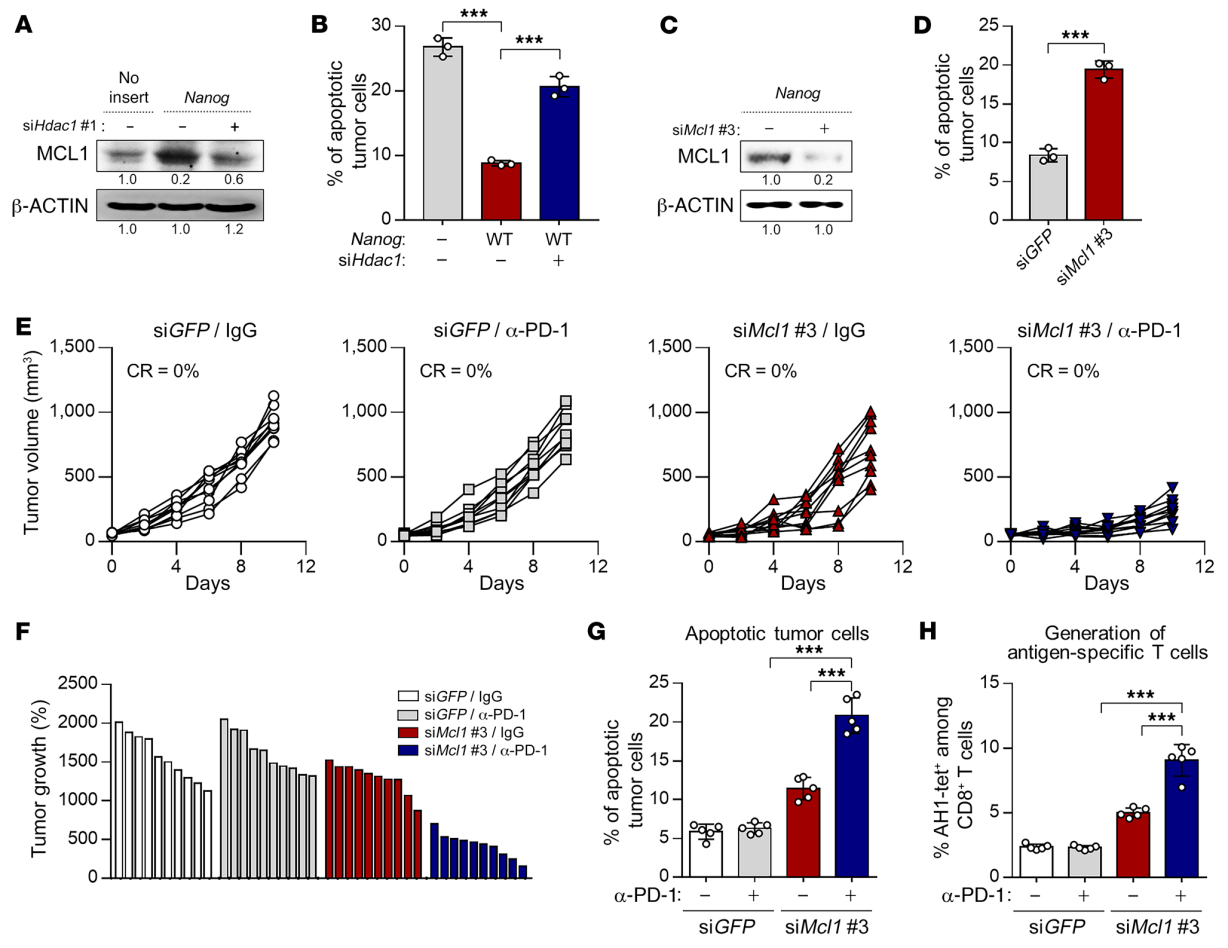
Resistance to CTL-mediated killing, regulated by the NANOG axis, is one of the key steps driving the immune-refractory feature of the TME. (**A** and **B**) CT26-no insert or CT26-*Nanog* cells were transfected with si*GFP* or si*Hdac1*. (**A**) Western blot analysis of MCL1 expression. β-Actin was used as an internal loading control. (**B**) Frequency of apoptotic cells. (**C** and **D**) CT26-*Nanog* cells were transfected with si*GFP* or si*Mcl1*. (**C**) Western blot analysis of the expression of MCL1. β-Actin was used as an internal loading control. (**D**) Frequency of apoptotic cells. (**E**–**H**) CT26-*Nanog* tumor–bearing mice were administered si*GFP* or si*Mcl1* with or without anti–PD-1 antibody treatment. (**E**) Tumor growth curves and (**F**) changes in tumor volume 17 days after challenge compared with baseline. (**G**) The frequency of apoptotic cells in the tumors. (**H**) Quantification of antigen-specific CTLs in spleens from tumor-bearing mice. Ten mice from each group were used for in vivo experiments. Results in the graphs represent 3 independent experiments performed in triplicate. Data represent the mean ± SD. ***P < 0.001, by 1-way ANOVA (**B**, **G**, and **H**) or 2-tailed Student’s *t* test (**D**).

Previous reports have indicated that tumor cell death by T cells is essential for reinvigorating the antitumor immunity cycle by providing tumor antigen stimuli to CTLs (41, 42, 52, 53), suggesting that the resistance of tumor cells to CTLs is a crucial obstacle to improving ICB therapy. In this regard, NANOG was also reported to induce resistance to CTL-mediated killing via HDAC1-mediated antiapoptotic protein MCL1 upregulation (28). Hence, we questioned whether the resistance to T cell–mediated killing induced by the NANOG/HDAC1/MCL1 axis is one of the crucial mechanisms triggering the immune-refractory feature in the TME by blocking the antitumor immunity cycle. To address this, we first assessed MCL1 expression levels in CT26-no insert-si*GFP*, CT26-*Nanog-*si*GFP*, and CT26-*Nanog-*si*Hdac1* cells. We found that MCL1 protein expression was substantially increased and that these increased levels of MCL1 mediated by NANOG were reversed upon HDAC1 silencing (Figure 5A). Consistent with the above results, we found that decreased CTL-mediated cell death by NANOG overexpression was reversed upon HDAC1 silencing (Figure 5B). Furthermore, MCL1 silencing reversed resistant phenotypes against the cognate CTLs of CT26-*Nanog* cells (Supplemental Figure 13 and Figure 5, C and D), suggesting a crucial role of MCL1 in the tumor-intrinsic CTL resistance induced by NANOG. Next, we treated CT26-*Nanog* tumor–bearing mice with intravenously administered chitosan nanoparticles carrying *Mcl1*- or *GFP*-targeting siRNA along with anti–PD-1, as illustrated in Supplemental Figure 14. We found that IgG- and anti–PD-1–treated CT26-*Nanog* tumors displayed similar growth rates under control si*GFP* treatment (Figure 5, E and F). However, si*Mcl1* treatment suppressed the growth of IgG and anti–PD-1–treated CT26-*Nanog* tumors. Of note, combined treatment with anti–PD-1 and si*Mcl1* drastically retarded tumor growth (Figure 5, E and F). In addition, we found that the percentage of apoptotic tumor cells was drastically increased by the combination treatment compared with treatment with either agent alone (Figure 5G). To test whether the generation of tumor-reactive T cells was affected by dual treatment, we isolated cells from the spleens of mice that received therapy and then assessed CT26-specific antigen AH1-tet^+^CD8^+^ T cells. Notably, AH1-tet^+^CD8^+^ T cells were increased in the combined treatment group compared with the other treatment groups (Figure 5H). However, similar to the results shown in Figure 4J, the combination treatment did not completely suppress tumor growth. Taken together, our data suggest that to completely overcome the resistance to anti–PD-1 therapy, the NANOG-mediated restriction mechanisms of the antitumor immunity cycle should be simultaneously blocked.

### The NANOG/HDAC1 axis controls T cell trafficking and resistance to CTL-mediated killing in multiple types of NANOG^hi^ tumor cells.

**Figure 6:**
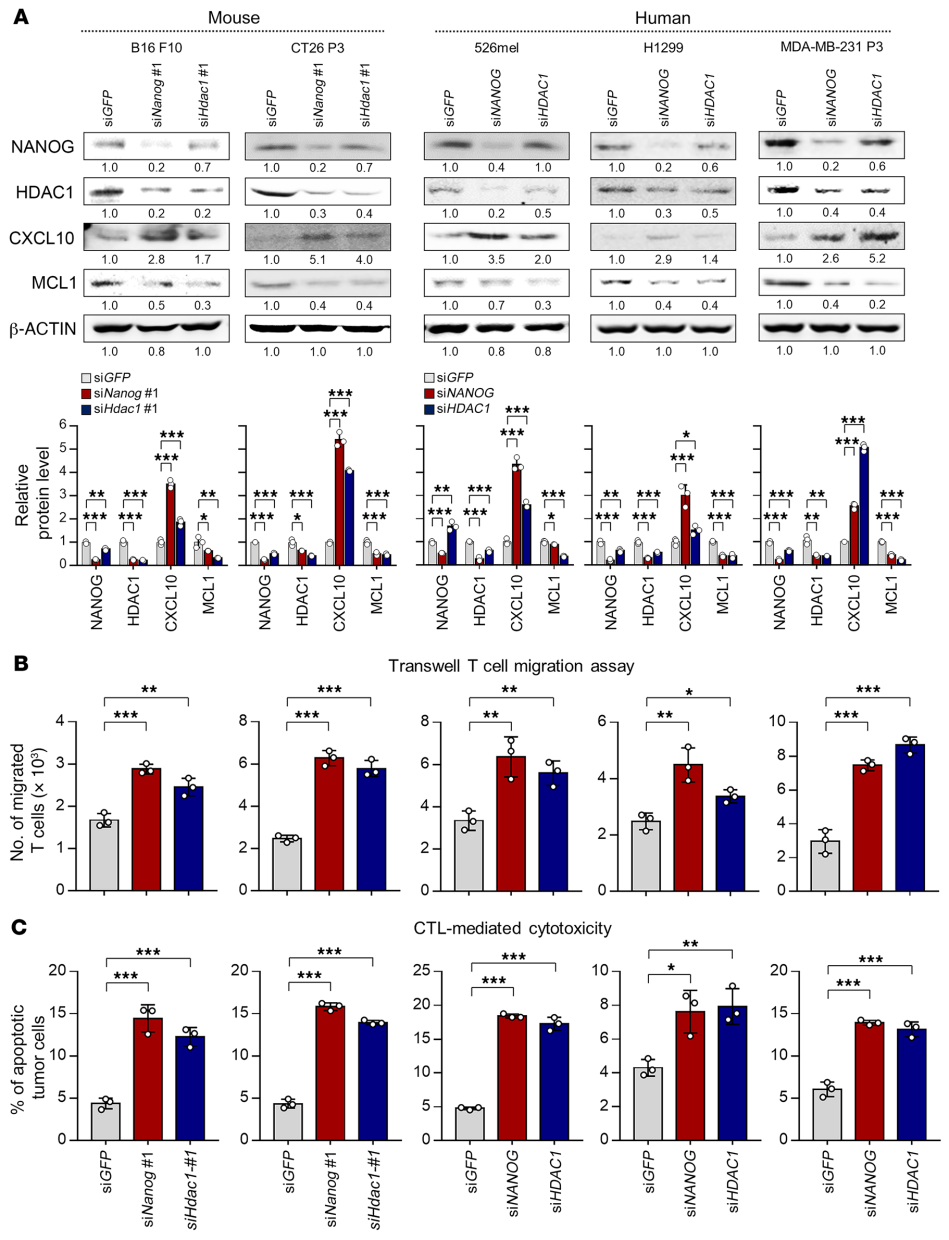
The NANOG/HDAC1 axis is conserved across multiple types of NANOG^hi^ tumor cells. (**A**–**C**) Various mouse or human cancer cell lines were transfected with si*GFP*, si*Nanog*, si*NANOG*, si*Hdac1*, or si*HDAC1*. (**A**) Western blot analysis of NANOG, HDAC1, CXCL10, and MCL1 expression. β-Actin was used as an internal loading control. Results in the graphs show the experimental quantitation based on at least 3 independent experiments. (**B**) Transwell T cell infiltration assay. (**C**) Frequency of apoptotic cells. Results in the graphs represent 3 independent experiments performed in triplicate. Data represent the mean ± SD. *P < 0.05, **P < 0.01, and ***P < 0.001, by 1-way ANOVA.

To verify the functional effects of the NANOG/HDAC1 axis in diverse types of mouse or human cancer cells, we further selected 3 NANOG-upregulated cancer cells, B16F10, 526mel, and H1299, and 3 NANOG^hi^ immunotherapeutic-refractory tumor models, MDA-MB-231 P3, CT26 P3, and YUMM2.1 P3, respectively (28). Knockdown of NANOG or HDAC1 with a siRNA robustly dampened expression of the effectors of NANOG signaling, such as CXCL10 and MCL1, across all tested cancer cells (Figure 6A and Supplemental Figure 15). Furthermore, si*Nanog*-, si*NANOG*-, si*Hdac1*-, or si*HDAC1*-treated tumor cells showed increased T cell infiltration and were more susceptible to CTL-mediated killing than were si*GFP*-treated tumor cells (Figure 6, B and C). These results demonstrate that the functional properties of the NANOG/HDAC1 axis are conserved across multiple types of cancer cells and that HDAC1 is an appropriate target for controlling immune-refractory NANOG^hi^ tumor cells.

### HDAC1 inhibition sensitizes NANOG^hi^-refractory tumor cells to anti–PD-1 therapy by reinvigorating the antitumor immunity cycle.

**Figure 7:**
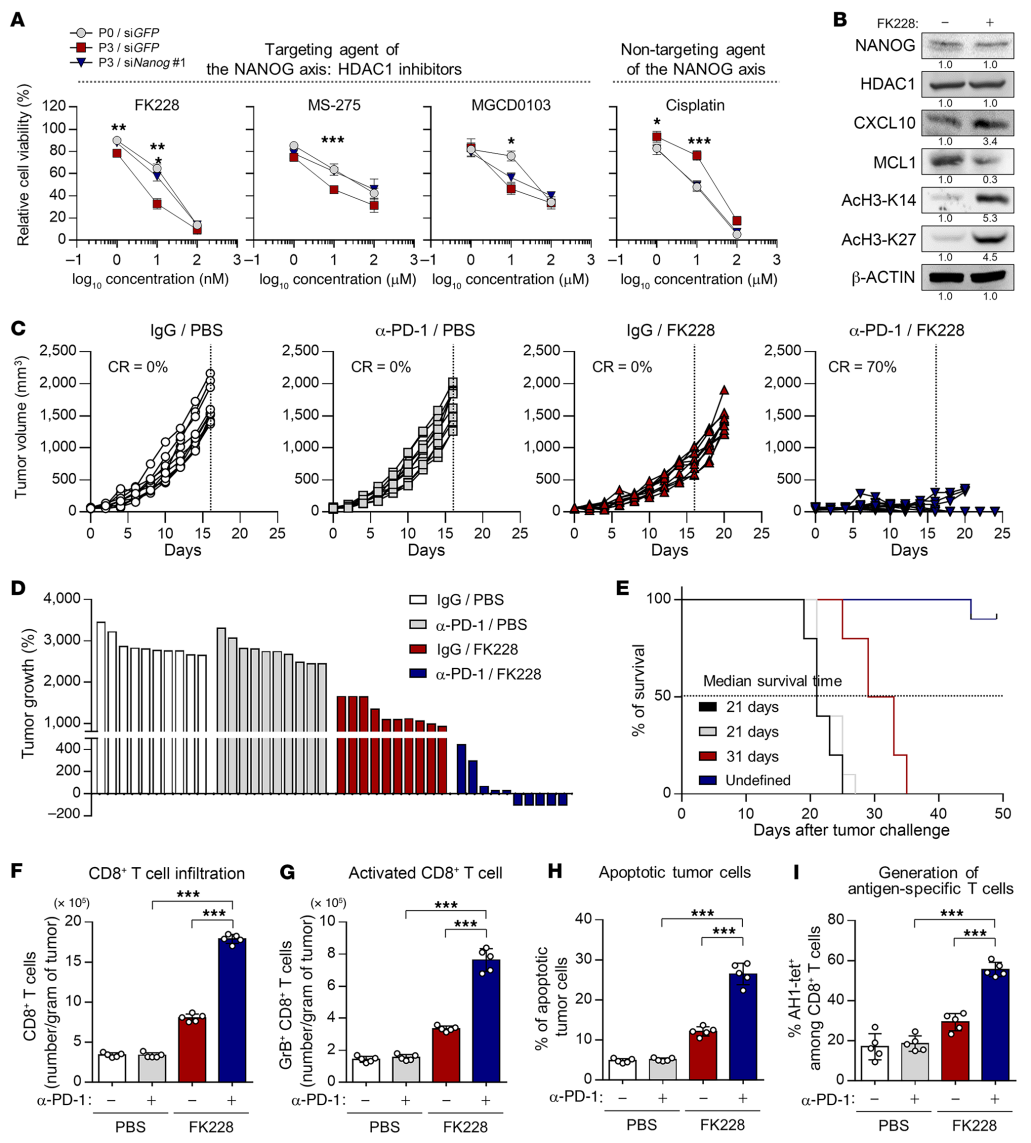
HDAC1 inhibition renders tumors susceptible to an anti–PD-1–mediated antitumor immune response. (**A**) CT26 P0 and CT26 P3 cells were transfected with siGFP or siNANOG. After 16 hours, cells were treated with the indicated concentrations of FK228, MS-275, SAHA, or cisplatin for 48 hours. Cell viability was measured by counting live cells using trypan blue. (**B**) Western blot analysis of NANOG, HDAC1, CXCL10, MCL1, AcH3-K14, and AcH3-K27 expression in CT26 P3 cells treated with DMSO or FK228. β-Actin was used as an internal loading control. (**C**–**I**) CT26 P3 tumor–bearing mice were administered vehicle or FK228, with or without PD-1 antibody treatment. (**C**) Tumor volume curves and (**D**) changes in tumor growth compared with baseline, 17 days after challenge. (**E**) Survival of mice inoculated with CT26 P3 and treated with the indicated reagents. (**F**) Flow cytometric profiles of tumor-infiltrating CD3^+^CD8^+^ T cells. (**G**) Ratio of granzyme B^+^ to tumor-infiltrating CD3^+^CD8^+^ T cells. (**H**) Frequency of apoptotic cells in the tumors. (**I**) Quantification of antigen-specific CTLs in spleens from tumor-bearing mice. Ten mice from each group were used for in vivo experiments. Results in the graphs represent 3 independent experiments performed in triplicate. Data represent the mean ± SD. *P < 0.05, **P < 0.01, and ***P < 0.001, by 2-way ANOVA (**A**) and 1-way ANOVA (**F**–**I**).

Although the data presented in this study demonstrate that targeting the NANOG/HDAC1 axis could be a promising approach for reversing the immune-refractory feature of the TME by simultaneously reinvigorating multiple steps of the antitumor immunity cycle, pharmacologic inhibitors of NANOG have yet to be developed. However, small-molecule inhibitors of HDAC1, such as FK228 (romidepsin), MS-275 (entinostat) or MGCD0103 (mocetinostat), have been used to treat patients with cancer (54–57). Therefore, to address the clinical applicability of HDAC1 inhibitors for controlling NANOG^hi^-refractory cancer, we first measured the viability of CT26 P0, CT26 P3, or CT26 P3-siNANOG cells after in vitro treatment with FK228, MS-275, or MGCD0103. We also used cisplatin as a control for the NANOG/HDAC1 axis–independent cancer drug. We found that CT26 P3 cells were more susceptible to HDAC1 inhibitors than were CT26 P0 cells and, conversely, that HDAC1 inhibitor sensitivity in CT26 P3 cells was reduced by knockdown of NANOG (Figure 7A). In contrast, the conventional drug cisplatin displayed the opposite phenomena (Figure 7A). Notably, among various HDAC1 inhibitors, FK228 was the most effective in CT26 P3 cells. We next assessed the expression of effectors for NANOG axis–mediated resistance to anti–PD-1 therapy. We found that HDAC1 inhibition by FK228 robustly dampened the expression of the effectors of resistance to anti–PD-1 therapy induced by the NANOG axis (Figure 7B and Supplemental Figure 16). These results suggest the possibility that targeting HDAC1 could reverse NANOG-mediated refractoriness to anti–PD-1 therapy.

To evaluate the preclinical therapeutic value of inhibiting HDAC1 and its downstream molecular axis, we tested the efficacy of anti–PD-1 therapy in BALB/c mice bearing CT26 P3 tumors. The mice received anti–PD-1 with FK228, according to the schedule described in Supplemental Figure 17. Tumors excised on day 20 were substantially smaller in size and tumor burden in the mice treated with both anti–PD-1 and FK228 compared with mice treated with either agent alone (Figure 7, C and D). Importantly, 90% of the mice treated with both anti–PD-1 and FK228 survived, even out to day 50 after tumor challenge. In contrast, all animals in the other groups had died by that point (Figure 7E). These results suggest that targeting HDAC1 could successfully reverse NANOG-mediated resistance to anti–PD-1.

We next assessed whether HDAC1 inhibition could switch the immune phenotype in the TME from an immune-refractory to an immune-stimulatory feature by reinvigorating the antitumor immunity cycle in NANOG^hi^-refractory tumor cells. We found that the overall numbers of CD8^+^ T cells and tumor-reactive CD8^+^ T cells expressing granzyme B were significantly higher in the combination treatment group than in the other treatment groups (Figure 7, F and G). Consistently, the percentage of apoptotic tumor cells and generation of tumor-specific CD8^+^ T cells were increased by the combined treatment compared with either treatment alone (Figure 7, H and I). Moreover, the above results were reproduced in the YUMM2.1 P3 tumor–bearing mice (Supplemental Figure 18). Taken together, our results demonstrate that HDAC1 inhibition switched the immune phenotypes from an immune-refractory to an immune-stimulatory feature by simultaneously reversing NANOG-mediated immune-refractory states, thereby overcoming the resistance to PD-1 blockade.

## Discussion

**Figure 8:**
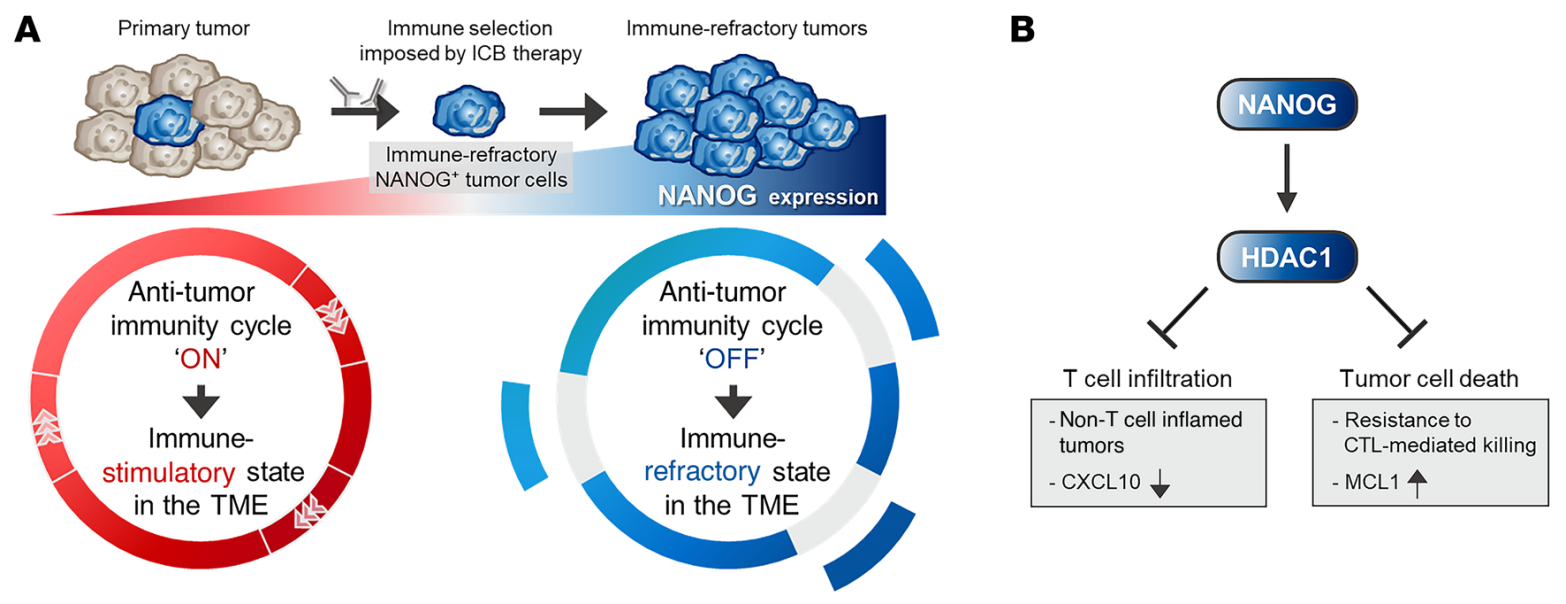
Model depicting the role of the NANOG/HDAC1 axis in resistance to anti–PD-1 therapy. (**A**) NANOG drives immune refractoriness against anti–PD-1 therapy by blocking the antitumor immunity cycle. (**B**) Molecular pathway through which the NANOG/HDAC1 axis represses T cell infiltration of tumors and tumor cell death by CTL-mediated killing.

The cancer immunoediting process occurs during the natural progression of tumors, but the available evidence from studies of patients treated with cancer immunotherapies indicates that this process reoccurs, either in part or in its entirety in response to treatment (7). Previous studies reported that immunoediting occurred not only close to the tumors refractory to various clinical interventions, including immunotherapy, but contributed to the generation of cancer cells with better survival advantages (7, 8). Notably, preferential selection and subsequent expansion of a subset of tumors by immunoediting leads to the evolution of tumors toward immune-refractory states (6–8). In this study, we showed that the immune-refractory states of tumor cells over the course of immunoediting process were closely linked to the immune-refractory feature of the TME accompanied by a simultaneous blockade of multiple steps of the antitumor immunity cycle via hyperactivation of the NANOG/HDAC1 axis (Figure 8, A and B). Further, to our knowledge, we are the first to report the role of the NANOG/HDAC1 axis in ICB therapy–refractory cancer.

The immunotherapy field has emphasized the targeting of inhibitory receptors expressed by immune cells (58). However, recent studies suggested that the engagement of tumor-intrinsic pathways in tumor cells is a critical mechanism by which these cells restrain the immunotherapy-driven antitumor immune response (16–19). A very important question is whether targeting tumor-intrinsic pathways is vital to extending the benefits of immunotherapy to a larger patient population, including those who have immune-refractory cancer. In this study, we demonstrated that NANOG expression in tumor cells provoked the immune-refractory feature in the TME by simultaneously disrupting various steps of the antitumor immunity cycle, such as T cell infiltration into tumors and T cell–mediated killing of tumor cells, in an HDAC1-dependent manner. Moreover, the suppression of NANOG expression in tumor cells converted the immune-refractory microenvironment into an immune-stimulatory microenvironment that facilitated antitumor immunity and overcame the resistance to anti–PD-1 therapy, as shown in Figure 2. Furthermore, repressing NANOG expression may represent a therapeutic strategy for initiating or reinvigorating an antitumor response in patients with anti–PD-1 therapeutic resistance. Together, our results indicate that NANOG, as a tumor-intrinsic factor, could be a critical determinant that confers resistance to anti–PD-1 therapy by determining the immune feature of the TME.

NANOG also works together with other stemness factors, such as MYC, OCT4, KLF4, SOX2, SOCS2, or ALDH1A1, to control a set of target genes that have important functions in embryonic stem cells and, plausibly, in tumor cells (59). Interestingly, results from previous studies indicated that these stemness factors including NANOG could induce immune evasion in various cancers by modulating the T cell–mediated antitumor response (60–62). To test whether other stemness factors may contribute to refractoriness to ICB therapy, we assessed the expression of these stemness factors in the transcriptomes of TCGA melanoma patients. Interestingly, not only NANOG but also MYC and SOCS2 were inversely associated with the gene signature that represents T cell infiltration and an antitumor response in our analysis of the patients (Supplemental Figure 19). These results suggest that, similar to NANOG, elevated MYC or SOCS2 expression in melanoma cells could shape the TME into expressing an immune-refractory feature, such as insufficient T cell trafficking to tumors and resistance of tumor cells to T cell–mediated killing. Therefore, it is worth understanding the relationship between the stemness factors and the immune-refractory feature of the TME for future studies.

As the NANOG/HDAC1 axis has been implicated as a central channel in the development of resistance to CTL-based immunotherapies, we believe that HDAC1 inhibition may be an effective strategy to control ICB therapy–refractory tumor cells with elevated expression of NANOG. Indeed, HDAC1 inhibition has received attention for therapeutic purposes in solid tumors and hematologic malignancies, even though HDAC1 inhibitors have shown limited responses as single agents in patients with cancer (54, 55). Recently, HDAC1 inhibition in combination with ICB therapy, as a therapeutic strategy aimed at converting non–T cell–inflamed into T cell–inflamed tumors, is currently being explored in multiple clinical trials (21, 63–66). Yet, only a minority of patients derives clinical benefit. Thus, it is important to identify the biomarker predicting the responses to HDAC1 inhibitors in combination with ICB therapy. In this study, we found that NANOG was associated with an immune-refractory feature of the TME and poor clinical outcomes in patients with cancer and that NANOG expression influenced the sensitivity of tumor cells to HDAC1 inhibitors. Therefore, these results show that NANOG can be used as a predictive biomarker allowing for the selection of patients who will receive clinical benefit from combined HDAC1 inhibitor and ICB therapy.

Altogether, we propose that NANOG^+^ immune-refractory tumor cells enriched by immune selection drive the immune-refractory feature of the TME by simultaneously disrupting multiple steps of the antitumor immunity cycle, which provokes resistance to PD-1 blockade (Figure 8, A and B). In this process, NANOG potentiates the resistance to anti–PD-1 therapy via HDAC1-mediated epigenetic reprogramming. Furthermore, we demonstrate that pharmacological inhibition of the NANOG/HDAC1 axis with FK228 triggered anti–PD-1 therapy–mediated tumor regression and made immunologically “cold” tumors “hot” by initiating or reinvigorating the antitumor immunity cycle. This could help to overcome the local immune-refractory environments seen in cancer and increase the effectiveness of classical ICB. Therefore, our results provide a strong rationale for the use of HDAC1 inhibitors as a promising strategy that we believe will be essential to extending the benefit of ICB therapy to larger patient populations, including those with NANOG^+^ immune-refractory tumors, particularly in immune-based cancer therapy.

## Methods

### Mice.

Six- to 8-week-old female BALB/c or male C57BL/6 mice were purchased from Central Lab Animal Inc.

### siRNA constructs.

The following synthetic siRNAs were produced by Bioneer: nonspecific *GFP*, 5′-GCAUCAAGGUGAACUUCAA-3′ (sense), 5′-UUGAAGUUCACCUUGAUGC-3′ (antisense); mouse *Nanog* no. 1, 5′-GCCUAGUUCUGAGGAAGCAUCGAAU-3′ (sense), 5′-AUUCGAUGCUUCCUCAGAACUAGGC-3′ (antisense); mouse *Nanog* no. 2, 5′-CCUCCAUUCUGAACCUGAGCUAUAA-3′ (sense), 5′-UUAUAGCUCAGGUUCAGAAUGGAGG-3′ (antisense); mouse *Nanog* no. 3, 5′-UGAACCUGAGCUAUAAGCAGGUUAA-3′ (sense), 5′-UUAACCUGCUUAUAGCUCAGGUUCA-3′ (antisense); human *NANOG*, 5′-GCAACCAGACCUGGAACAA-3′ (sense), 5′-UUGUUCCAGGUCUGGUUGC-3′ (antisense); mouse *Hdac1* no. 1, 5′-GCAUGACUCACAAUUUGCUGCUCAA-3′ (sense), 5′-UUGAGCAGCAAAUUGUGAGUCAUGC-3′ (antisense); mouse *Hdac1* no. 2, 5′-UGUCCGGUGUUUGAUGGCUUGUUUG-3′ (sense), 5′-CAAACAAGCCAUCAAACACCGGACA-3′ (antisense); mouse *Hdac1* no. 3, 5′-CCAUGCAAAGAAGUCUGAAGCUUCU-3′ (sense), 5′-AGAAGCUUCAGACUUCUUUGCAUGG-3′ (antisense); human *HDAC1*, GAGUCAAAACAGAGGAUGA-3′ (sense), 5′-UCAUCCUCUGUUUUGACUC-3′ (antisense); mouse *Mcl1* no. 1, 5′-GGGCAGGAUUGUGACUCUUAUUUCU-3′ (sense), 5′-AGAAAUAAGAGUCACAAUCCUGCCC-3′ (antisense); mouse *Mcl1* no. 2, 5′-GGCAGGAUUGUGACUCUUAUUUCUU-3′ (sense), 5′-AAGAAAUAAGAGUCACAAUCCUGCC-3′ (antisense); and mouse *Mcl1* no. 3, 5′-GCAGGAUUGUGACUCUUAUUUCUUU-3′ (sense), 5′-AAAGAAAUAAGAGUCACAAUCCUGC-3′ (antisense).

### Cell lines and reagents.

CT26, B16F10, 526mel, H1299, and MDA-MB-231 cell lines were purchased from the American Type Culture Collection (ATCC). All cell lines were obtained between 2010 and 2014. The YUMM2.1 cell lines were generated in-house in 2021. The cells were tested for mycoplasma using a Mycoplasma Detection Kit (Thermo Fisher Scientific). The identities of the cell lines were confirmed by short tandem repeat (STR) profiling by IDEXX Laboratories Inc. and used within 6 months. To generate ICB therapy–resistant tumor cells, BALB/c or C57BL/6 mice were subcutaneously inoculated with 1 × 10^5^ CT26 (CT26 P0) or 1 × 10^6^ YUMM2.1 (YUMM2.1 P0) cells per mouse, respectively. Between 5 and 7 days of the tumor challenge, mice were treated with anti–PD-1 antibody (200 μg; BioXcell) three times per week. This treatment regimen was repeated for 2 cycles. The surviving P0 cells were termed P1 cells. This process was repeated for 3 cycles to derive the P3 line, which was impervious to therapeutic effect by anti–PD-1. As a control, we performed this procedure using anti-IgG antibody in mice inoculated with P0 cells to generate N1, N2, and N3 cells without immune selection. MDA-MB-231 P3 cell lines have been previously described (28). To generate CT26-*NANOG* and CT26-*NANOG*-*CXCL10* cells, pMSCV-*NANOG* or pMSCV-*CXCL10*-*GFP* plasmids were first transfected along with viral packaging plasmids (VSVG and Gag-pol) into HEK293FT cells. Three days after transfection, the viral supernatant was filtered through a 0.45 μm filter and used to infect CT26 or CT26-*NANOG* cells. The infected cells were then selected with 1 μg/mL puromycin (CT26-*NANOG*) or sorted for GFP^+^ cells by flow cytometry (CT26-*NANOG*-*CXCL10*). All cells were grown at 37°C in a 5% CO_2_ incubator/humidified chamber. Cisplatin, FK228, MS-275, and MGCD0103 were purchased from Selleckchem.

### DNA constructs.

The pMSCV-*NANOG* WT and MT plasmids have been described previously (9, 10). Briefly, to generate pMSCV/*Nanog*, DNA fragments encoding Nanog were amplified from pSIN-EF2-Nanog-Pur–expressing cells (Addgene) using the following primer set: 5′-GCCTCGAGATGAGTGTGGATCCAGCTTG-3′ and 5′-GCGAATTCTCACACGTCTTGAGGTTG-3′. Amplified DNA was subcloned into the XhoII/EcoRI site of the pMSCV retroviral vector (Clontech). To create mutations in the *Nanog* gene, the QuikChange XL Site-Directed Mutagenesis Kit was used (Stratagene). Plasmid integrity was verified by DNA sequencing. pMSCV-*CXCL10* WT-*GFP* and MT-*GFP* plasmids were purchased from Cosmogenetech.

### Real-time qPCR.

The experimental procedure has been described previously (67). Briefly, total RNA was extracted with the RNeasy Mini Kit (QIAGEN) and treated with DNase (Thermo Fisher Scientific). qPCR mixtures were assembled with 1 μL cDNA template, iQ SYBR Green Supermix (Bio-Rad), and primers for *Cxcl9* or *Cxcl10*. The following qPCR primers were purchased from Bioneer: *Cxcl9*, 5′-CGAGGCACGATCCACTACAA-3′ (forward), 5′-AGGCAGGTTTGATCTCCGTT-3′ (reverse); and *Cxcl10*, 5′-ATGACGGGCCAGTGAGAATG-3′ (forward), 5′-TCAACACGTGGGCAGGATAG-3′ (reverse). PCR was carried out for 40 cycles with the following thermal cycling conditions: 95°C for 10 seconds (denaturation) and 61°C for 60 seconds (annealing). All data were normalized to *Actb* mRNA expression levels.

### Western blot analysis.

Lysate extracted from 1 × 10^5^ cells was used to perform Western blotting. The following primary antibodies were used: anti-NANOG (A300-379Am Bethyl Laboratories); anti-FLAG (catalog M185-3L, MBL International); anti-HDAC1 (catalog 5356S, Cell Signaling Technology); anti-AcH3K14 (catalog 4318P, Cell Signaling Technology); anti-AcH3K27 (catalog 4353P, Cell Signaling Technology); anti-MCL1 (catalog sc-819, Santa Cruz Biotechnology); anti-CXCL10 (catalog 551215, BD Biosciences); and anti–β-actin (catalog M177-3, MBL International). Western blotting was followed by incubation with the appropriate secondary antibodies conjugated to HRP. The immunoreactive bands were developed with the chemiluminescence ECL Detection system (GE Healthcare), and signals were detected using a luminescence image analyzer (LAS-4000 Mini, Fujifilm).

### ChIP and quantitative ChIP assays.

The ChIP kit (MilliporeSigma) was used according to the manufacturer’s instructions. Briefly, cells (1 × 10^7^ per assay) were bathed in 1% formaldehyde at 25°C for 10 minutes to crosslink proteins and DNA and then lysed in SDS buffer containing protease inhibitors. DNA was sheared by sonication using a Sonic Dismembrator Model 500 (Thermo Fisher Scientific). Immunoprecipitation was carried out by incubation with 1 μg anti-HDAC1 antibodies (catalog 5356S, Cell Signaling Technology) or rabbit anti-IgG (MilliporeSigma) for 16 hours. For the quantitative ChIP (qChIP) assay, immunoprecipitated DNA was quantified by real-time qPCR using the following primer sets: 5′- CACTGTCACCTCTATGCGAGAT-3′ (forward) and 5′-CACTCTGCACAGCACCCAAG-3′ (reverse). Each sample was assayed in triplicate, and the amount of precipitated DNA was calculated as the percentage of the input sample.

### CTL-mediated apoptosis assay.

Tumor cells were labeled with CFSE (10 μM, Molecular Probes) in RPMI supplemented with 0.1% FBS. The CFSE-labeled CT26 cells were mixed with cognate AH1–specific CD8^+^ CTLs at a 1:1 ratio and incubated for 4 hours at 37°C. The cells were stained for active caspase-3 as an index of apoptosis and examined by flow cytometry.

### Granzyme B–mediated apoptosis assay.

Recombinant human granzyme B (Enzo Life Sciences) was mixed with BioPorter Reagent (MilliporeSigma) at 25°C for 5 minutes. The tumor cells were mixed with BioPorter–granzyme B complexes for 4 hours at 37°C. Next, the cells were stained for active caspase-3 as an index of apoptosis and examined by flow cytometry.

### Trypan blue exclusion assay.

To determine cell viability, a trypan blue exclusion assay was performed. Briefly, cells were seeded at 1 × 10^5^ cells per well in 12-well plates 1 day prior to the assay. The treatments were added at the concentrations indicated in the figures. After 24 hours, the cells were detached and stained with 0.4% trypan blue. Unstained cells were counted using a hemocytometer. The data are expressed as the percentages of unstained cells compared with the control cells not exposed to the chemical reagents.

### TCGA data collection and analysis.

Gene expression data for more than 10,000 cancer samples profiled by TCGA were collected from the Firehose BROAD GDAC data repository (https://gdac.broadinstitute.org/). Clinical data were also retrieved from the same source. The T cell infiltration and T cell–mediated antitumor response gene expression signatures were previously defined (29–32). We used the single-sample gene set enrichment analysis (GSEA) algorithm implemented in R package’s gene set variation analysis (GSVA) to calculate the T cell infiltration or antitumor response signature scores for each sample. The default parameters from the GSVA package were used. Spearman’s correlation was used to quantify the association between NANOG signature, T cell infiltration, and antitumor response scores individually for each tumor type. The association between NANOG signature expression and survival was evaluated by Cox regression and Kaplan-Meier analyses. For the latter, the samples were stratified into 3 groups according to their NANOG signature expression (low, intermediate, high), and the 20th and 80th percentiles were used as cutoff thresholds.

### Tumor treatment experiments.

BALB/c mice were inoculated subcutaneously with 1 × 10^5^ CT26 P3 cells per mouse. Seven days after tumor challenge, FK228 (0.05 mg/kg) or PBS was administered via the intraperitoneal route. The day after FK228 treatment, mice were administered anti–PD-1 (BioXcell) or an isotype control antibody given every 3 days at a dose of 200 μg per mouse in accordance with the schedule described in Supplemental Figure 16. This treatment regimen was repeated for 3 cycles. The mice were monitored for tumor burden and survival for 20 days and 50 days after challenge, respectively.

### Tumor digestion, cell isolation, and flow cytometric analysis.

The treated mice were euthanized on day 18 following tumor inoculation, and tumors were harvested. The tumors were dissected into fragments by cutting and digested by collagenase (0.5 mg/mL, MilliporeSigma) and DNase (1 μg/mL, MilliporeSigma) at 37°C for 45 minutes. The digested samples were then filtered through a 70 μm cell strainer and washed with PBS buffer. The cell pellets were incubated with RBC lysis buffer to lyse the RBCs. The cell suspensions were stained for the intracellular and extracellular protein markers of interest, and the stained samples were assessed on a flow cytometer (BD biosciences) along with CellQuest Pro software. The following staining antibodies used: anti-CD3, anti-CD4, anti-CD19, anti-Foxp3, anti-CD8, anti–granzyme B, anti–active caspase-3, and anti–IFN-γ (all from BD Biosciences).

### Data availability.

Transcriptomic data from patients with melanoma classified as responders or nonresponders to anti–PD-1 treatment are available in the NCBI’s Gene Expression Omnibus (GEO) database (GEO GSE91061; https://www.ncbi.nlm.nih.gov/geo/query/acc.cgi?acc=GSE91061; Riaz et al. cohort); the European Nucleotide Archive (ENA) (PRJEB23709; https://www.ebi.ac.uk/ena/browser/view/PRJEB23709; Gide et al. cohort); and the Database of Genotypes and Phenotypes (dbGaP) (phs000452.v3.p1; https://www.ncbi.nlm.nih.gov/projects/gap/cgi-bin/study.cgi?study_id=phs000452.v3.p1; Liu et al. cohort). Transcriptomic data from TCGA were deposited in the Firehose data repository portal (https://gdac.broadinstitute.org/). The gene ontology (GO) analysis, which supported the findings of this study, is publicly available online in the Enrichr database (https://amp.pharm.mssm.edu/Enrichr/). The raw images for the immunoblots are provided in the supplemental materials.

### Statistics.

All data are representative of at least 3 separate experiments. Statistical differences were calculated by Student’s *t* test (2-tailed, unpaired), 1-way ANOVA, or 2-way ANOVA using GraphPad Prism (GraphPad Software). Spearman’s rank correlation analysis was used to evaluate the association between the indicators. Survival curves were calculated using the Kaplan-Meier method, and the differences between the survival curves were calculated by the long-rank test. A Cox proportional hazards model was created to identify the independent predictors of survival. Results with 2-tailed *P* values of less than 0.05 were considered statistically significant.

### Study approval.

All mice were maintained and handled under a protocol approved by the IACUC of Korea University (KOREA-2017-0141, Seoul, South Korea).

## Author contributions

SJO, HJL, KHS, and TWK designed the study. SJO, HJL, KHS, SK, and EC conducted the experiments. SJO, HJL, and KHS acquired and analyzed data. MWB provided experimental materials. SJO, JL, and TWK wrote the manuscript. All authors read and approved the final manuscript.

## Supplementary Material

Supplemental data

## Version 1 02/01/2022

In-Press Preview

## Version 2 03/15/2022

Electronic publication
