# Supplementary material for: Targeting the NANOG/HDAC1 axis reverses resistance to PD-1 blockade by reinvigorating the antitumor immunity cycle
Source: J Clin Invest. 2022 Mar 15;132(6):e147908. doi: 10.1172/JCI147908 (PMC8920337; doi:10.1172/JCI147908)

Supplemental information for the manuscript entitled:

Targeting NANOG/HDAC1 axis reverses resistance to PD-1 blockade by reinvigorating anti-tumor immunity cycle

Se Jin Oh. et al.,

Supplemental Figure 1. Anti-tumor immune state signature could predict response to anti-PD-1 therapy.

Supplemental Figure 2. NANOG is inversely associated with anti-tumor immune state of the TME in melanoma patients treated with anti-PD-1.

Supplemental Figure 3. Diagram depicting the process of *in vivo* immune selection by anti-PD-1 therapy.

Supplemental Figure 4. Diagram depicting the schedule of *in vivo* treatment.

Supplemental Figure 5. YUMM2.1 P3 cells display the immune-refractory feature of the TME.

Supplemental Figure 6. MHC class I expression and CTL activation capacity of tumor cells are unaffected by immune selection.

Supplemental Figure 7. CT26 P3 cells are more resistant to lysis by antigen-specific CTLs or granzyme B compared with P0 cells.

Supplemental Figure 8. NANOG is overexpressed in YUMM2.1 P3 cells compared to YUMM2.1 P0 cells.

Supplemental Figure 9. Test of siRNA-targeting *Nanog* to investigate the role of NANOG in immunotherapeutic resistance.

Supplemental Figure 10. Diagram depicting the schedule of *in vivo* treatment.

Supplemental Figure 11. NANOG reduces response to anti-PD-1 therapy by inducing immuno-refractory state in the TME.

Supplemental Figure 12. Test of siRNA-targeting *Hdac1* to investigate the role of HDAC1 in immunotherapeutic resistance.

Supplemental Figure 13. Test of siRNA-targeting *Mcl1* to investigate the role of MCL1 in immunotherapeutic resistance.

Supplemental Figure 14. Diagram depicting the schedule of *in vivo* treatment.

Supplemental Figure 15. The NANOG/HDAC1 axis is conserved in YUMM2.1 P3 cells.

Supplemental Figure 16. HDAC1 inhibition by FK228 reduces expression of the effectors of ICB therapy-refractoriness induced by the NANOG axis in YUMM2.1 P3

Supplemental Figure 17. Diagram depicting the schedule of *in vivo* treatment.

Supplemental Figure 18. Targeting HDAC1 overcomes resistance to anti-PD-1 therapy in YUMM2.1 P3

Supplemental Figure 19. MYC and SOCS2 are inversely associated with immune-refractory feature of the TME in melanoma patients.

Supplemental Table 1. Differentially expressed genes between NANOG^{high} and NANOG^{low} patients.

Supplemental Figures and Figure legends.

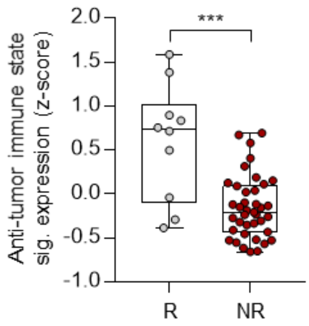

Supplemental Figure 1. Anti-tumor immune state signature could predict response to anti-PD-1 therapy. Comparisons of expression level of anti-tumor immune state signatures genes in responders (R, *n* = 10), and non-responders (NR, *n* = 39) to PD-1 blockade therapy. The p-values were determined by unpaired, two-tailed Student's t-test. In the box plots, the top and bottom edges of the boxes indicate the first and third quartiles, respectively; the center lines indicate the medians, and the ends of the whiskers indicate the maximum and minimum values. Source data are provided as a Source Data file. (*P ≤ 0.05, **P ≤ 0.01, ***P ≤ 0.001. NS, not significant)

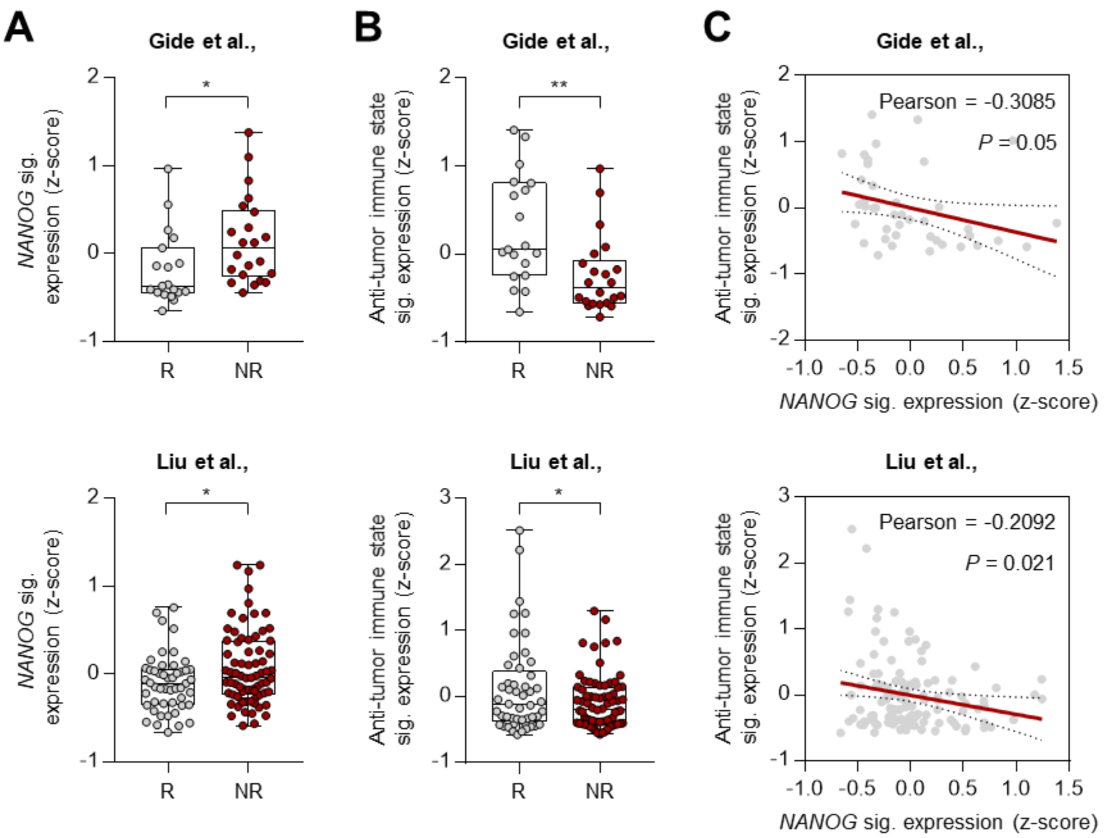

Supplemental Figure 2. NANOG is inversely associated with anti-tumor immune state of the TME in melanoma patients treated with anti-PD-1. (**A** to **C**) Top, indicated signature expression or correlation of responder (R, *n* = 19) and non-responders (NR, *n* = 22) in Gide et al., cohort. Bottom, indicated signature expression or correlation of responder (R, *n* = 49) and non-responders (NR, *n* = 73) in Liu et al., cohort. (**A** and **B**) Comparisons of the expression levels of indicated signature. (**C**) Pearson`s correlation between expression level of NANOG signature and anti-tumor immune states in melanoma patients treated with anti-PD-1. Correlation and two-tailed p-values were assessed using the Pearson's correlation coefficient and the unpaired t-test. The p-values were determined by unpaired, two-tailed Student's t-test. In the box plots, the top and bottom edges of boxes indicate the first and third quartiles, respectively; the center lines indicate the medians; and the ends of the whiskers indicate the maximum and minimum values, respectively, Source data are provided as a Source Data file. (*P ≤ 0.05, **P ≤ 0.01, ***P ≤ 0.001. NS, not significant).

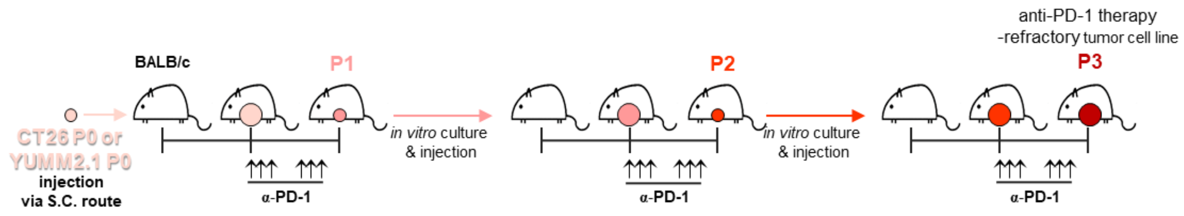

Supplemental Figure 3. Diagram depicting the process of *in vivo* immune selection by anti-PD-1 therapy. Refractory tumor model to anti-PD-1 therapy were generated from an anti-PD-1-susceptible parental cell line CT26 P0 or YUMM2.1 P0.

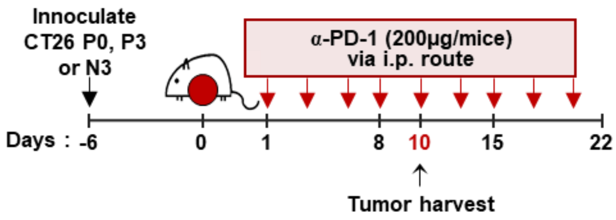

Supplemental Figure 4. Diagram depicting the schedule of *in vivo* treatment. Schematic of the therapy regiment in BALB/c mice implanted with CT26 P0, CT26 P3 or CT26 N3 cells were treated with indicated reagents.

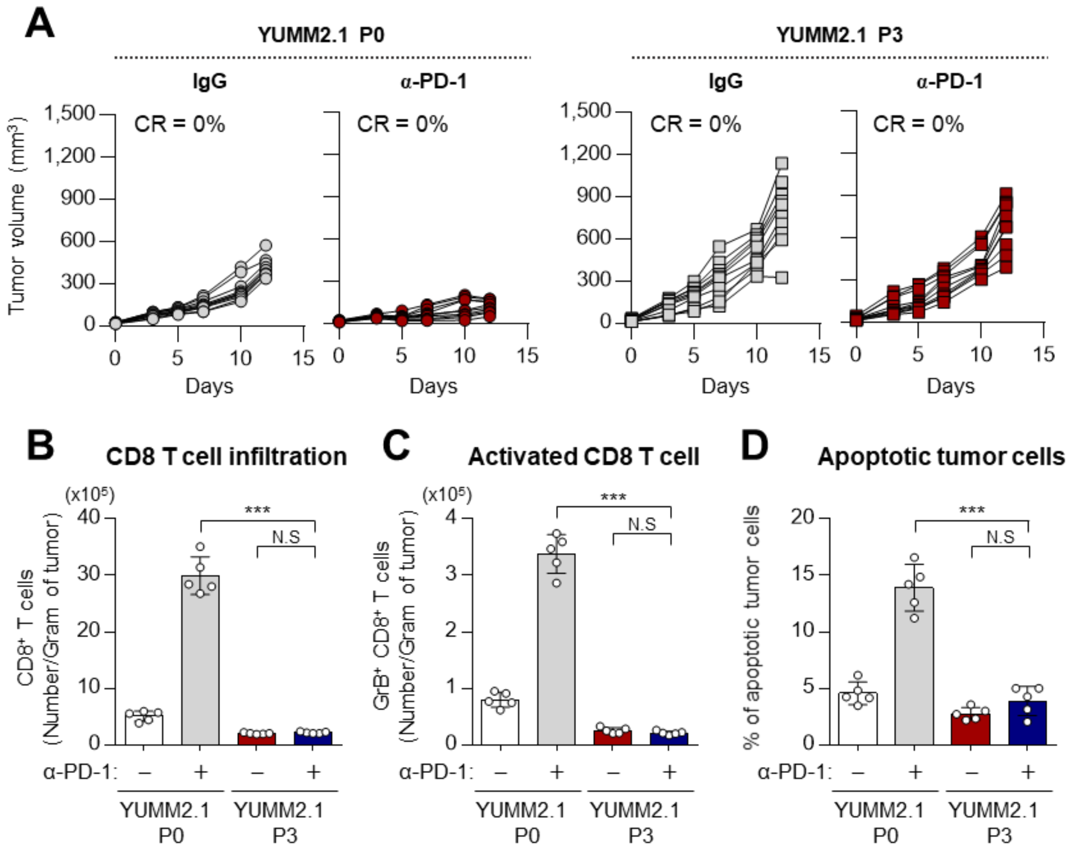

Supplemental Figure 5. YUMM2.1 P3 cells display the immune-refractory feature of the TME. (**A** to **D**) YUMM2.1 P0 or P3 tumor-bearing mice treated with IgG or PD-1 antibody. (**A**) Tumor growth curves. (**B**) Flow cytometry profiles of the tumor-infiltrating $CD3^+$ $CD8^+$ T cells. (**C**) The ratio of granzyme B^+ to tumor-infiltrating $CD3^+$ $CD8^+$ T cells. (**D**) The frequency of apoptotic cells in the tumors. Ten mice from each group were used for in vivo experiments. The graphs represent three independent experiments performed in triplicate. (**B** to **D**) The p-values by one-way ANOVA are indicated. The data represent the mean ± SD. Source data are provided as a Source Data file. (*P ≤ 0.05, **P ≤ 0.01, ***P ≤ 0.001. NS, not significant)

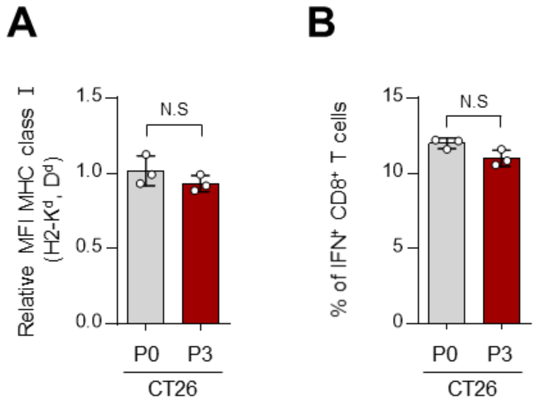

Supplemental Figure 6. MHC class I expression and CTL activation capacity of tumor cells are unaffected by immune selection. (**A**) MHC class I expression on CT26 P0 or P3 cells was measured by flow cytometry. (**B**) CT26 P0 or P3 cells were incubated with AH1-specific T cells at a 1:1 effector : target ratio for 16 hours. The cells were then stained for surface CD8 and intracellular IFN-γ to detect CTL activation. The graphs represent three independent experiments performed in triplicate. The p-values were determined by unpaired, two-tailed Student's t-test. The data represent the mean ± SD. Source data are provided as a Source Data file.

(*P ≤ 0.05, **P ≤ 0.01, ***P ≤ 0.001. NS, not significant)

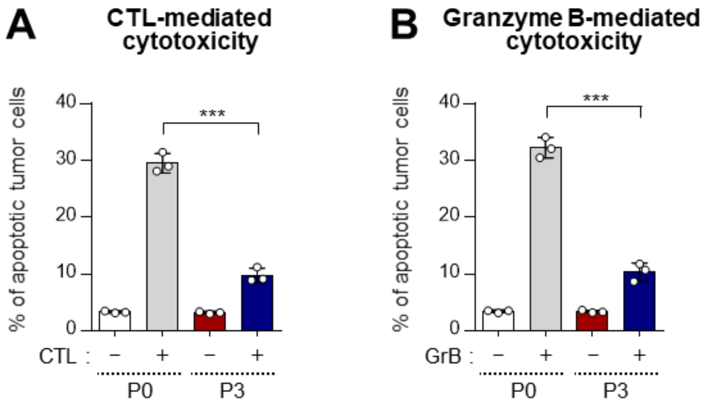

Supplemental Figure 7. CT26 P3 cells are more resistant to lysis by antigen-specific CTLs or granzyme B compared with P0 cells. (**A**) The frequency of apoptotic (active caspase-3+) cells in CT26 P0 or P3 cells after incubation with or without AH1-specific CTLs at a 1:1 ratio for 4 hours was estimated by flow cytometry analysis. (**B**) The frequency of apoptotic (active caspase-3+) cells in CT26 P0 or P3 cells after incubation with or without Granzyme B for 6 hours was analyzed by flow cytometry. The graphs represent three independent experiments performed in triplicate. The p-values by one-way ANOVA are indicated. The data represent the mean ± SD. Source data are provided as a Source Data file. (*P ≤ 0.05, **P ≤ 0.01, ***P ≤ 0.001. NS, not significant)

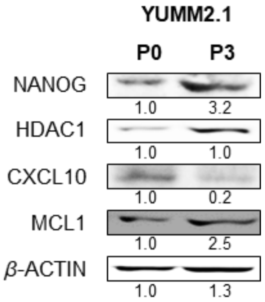

Supplemental Figure 8. NANOG is overexpressed in YUMM2.1 P3 cells compared to YUMM2.1 P0 cells. Western blot analysis of the expression of NANOG, HDAC1, CXCL10 and MCL1. β-actin was used as an internal loading control.

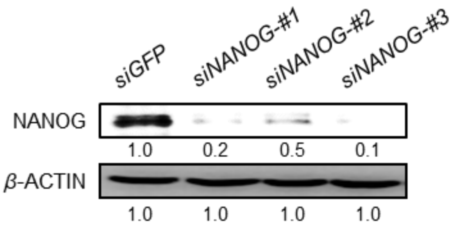

Supplemental Figure 9. Test of siRNA-targeting *Nanog* to investigate the role of NANOG in immunotherapeutic resistance. CT26 P3 cells were transfected with *siGFP* or *siNanog-#1, -#2, -#3*. Protein level of NANOG was analyzed by immunoblotting. β-ACTIN was included as an internal loading control. Numbers below blot images indicate the expression as measured by fold change.

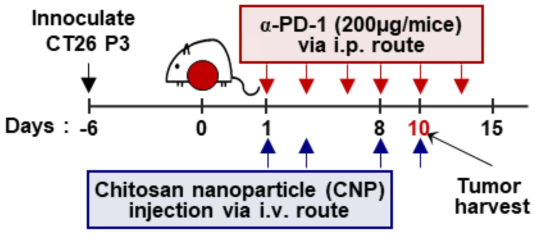

Supplemental Figure 10. Diagram depicting the schedule of *in vivo* treatment. Schematic of the therapy regiment in BALB/c mice implanted with CT26 P3 cells were treated with the indicated reagents.

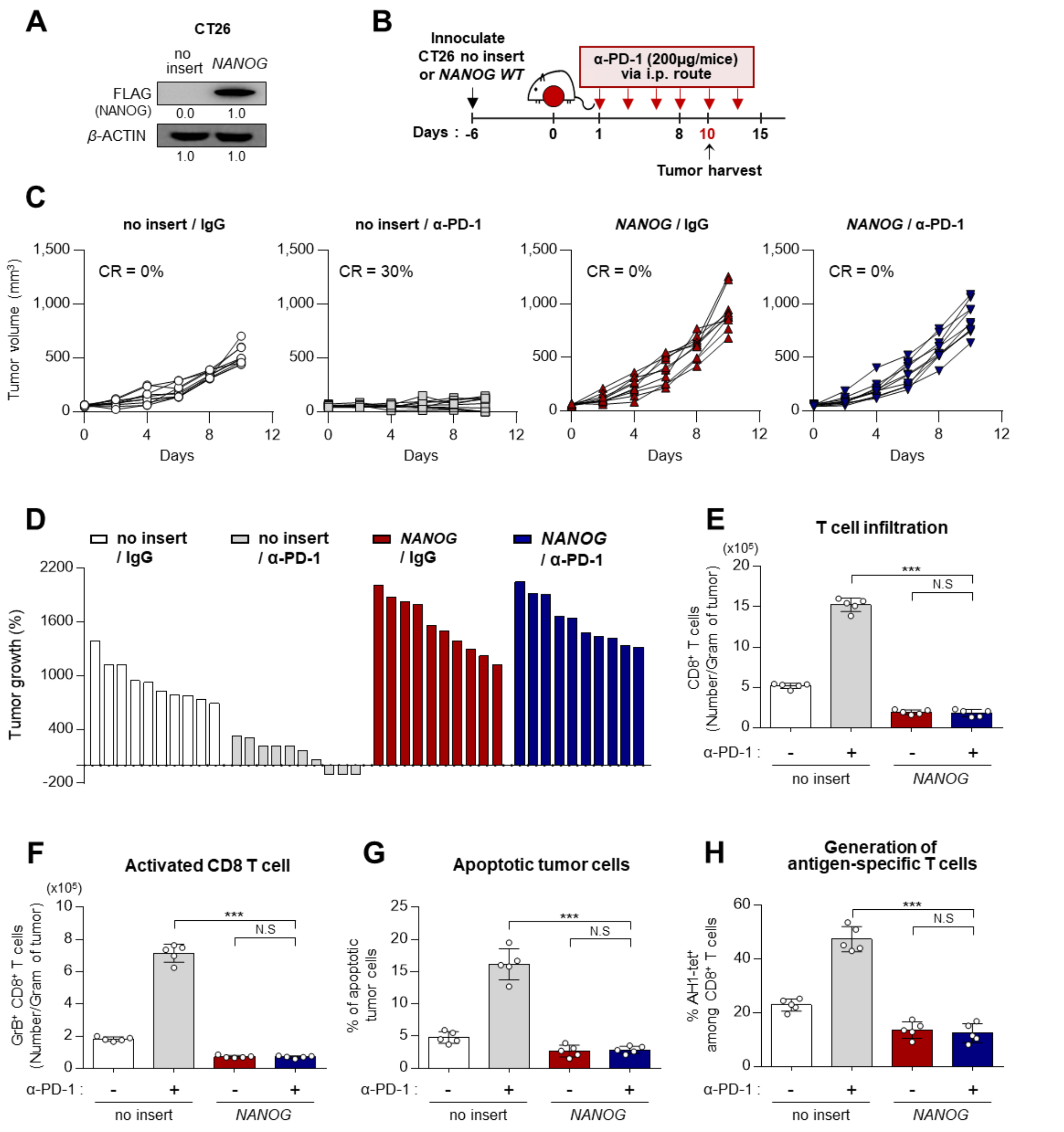

Supplemental Figure 11. NANOG reduces response to anti-PD-1 therapy by inducing immuno-refractory state in the TME. (**A**) CT26 P0 cells were stably transfected with empty vector (no insert) or *Nanog*. Protein level of NANOG was analyzed by Western blot. (**B**) Schematic of the therapy regiment in BALB.c mice implanted with CT26 no insert or *Nanog* cells were treated with the indicated reagents. (**C** to **H**) CT26-no insert or CT26-*Nanog* tumor-bearing mice were treated with IgG or PD-1 antibody. (**C**) Tumor volume and (**D**) percentage of tumor growth at 17 days after challenge. (**E**) Flow cytometry profiles of tumor-infiltrating CD8$^+$ T cells. (**F**) The absolute number of GrB$^+$ tumor-infiltrating CD8$^+$ T cells. (**G**) The frequency of apoptotic cells in the tumors treated with the indicated reagents. (**H**)

The percentage of AH1-specific T cells (AH1-tetramer^{+}) among CD8^{+} T cells were analyzed by flow cytometry. Numbers below blots indicate the expression as measured by fold change. Ten mice from each group were used for in vivo experiments. The graphs represent three independent experiments performed in triplicate. (**E** to **H**) The p-values by one-way ANOVA are indicated. The data represent the mean ± SD. Source data are provided as a Source Data file. (*P ≤ 0.05, **P ≤ 0.01, ***P ≤ 0.001. NS, not significant)

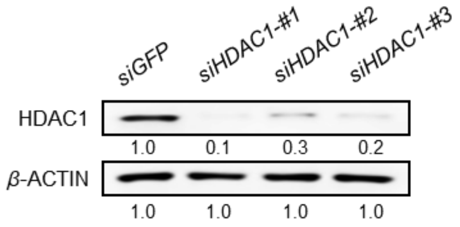

Supplemental Figure 12. Test of siRNA-targeting *Hdac1* to investigate the role of HDAC1 in immunotherapeutic resistance. CT26 P3 cells were transfected with *siGFP* or *siHdac1-#1, -#2, -#3*. Protein level of HDAC1 was analyzed by immunoblotting. β-ACTIN was included as an internal loading control. Numbers below blot images indicate the expression as measured by fold change.

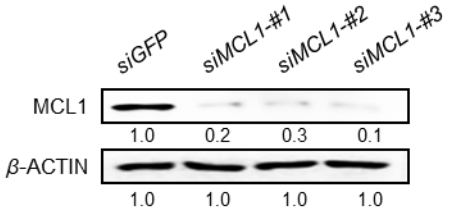

Supplemental Figure 13. Test of siRNA-targeting *Mcl1* to investigate the role of MCL1 in immunotherapeutic resistance. CT26 P3 cells were transfected with *siGFP* or *siMcl1-#1, -#2, -#3*. Protein level of MCL1 was analyzed by immunoblotting. β-ACTIN was included as an internal loading control. Numbers below blot images indicate the expression as measured by fold change.

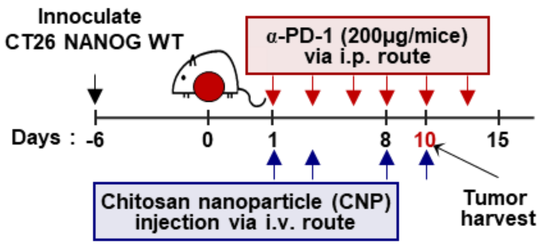

Supplemental Figure 14. Diagram depicting the schedule of *in vivo* treatment. Schematic of the therapy regiment in BALB/c mice implanted with CT26 NANOG WT cells were treated with the indicated reagents.

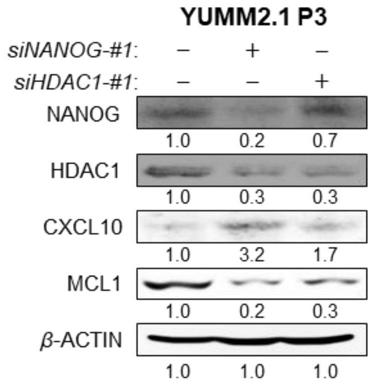

Supplemental Figure 15. The NANOG/HDAC1 axis is conserved in YUMM2.1 P3 cells. YUMM2.1 P3 cells were transfected with *siGFP*, *siNanog* or *siHdac1*. Western blot analysis of NANOG, HDAC1, CXCL10, and MCL1 expression. β-actin was used as an internal loading control. Numbers below blot images indicate the expression as measured by fold change.

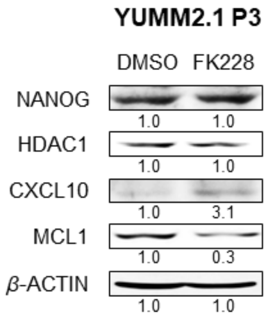

Supplemental Figure 16. HDAC1 inhibition by FK228 reduces expression of the effectors of ICB therapy-refractoriness induced by the NANOG axis in YUMM2.1 P3. YUMM2.1 P3 cells treated with FK228 or DMSO. Western blot analysis of NANOG, HDAC1, CXCL10, and MCL1 expression. β-actin was used as an internal loading control. Numbers below blot images indicate the expression as measured by fold change.

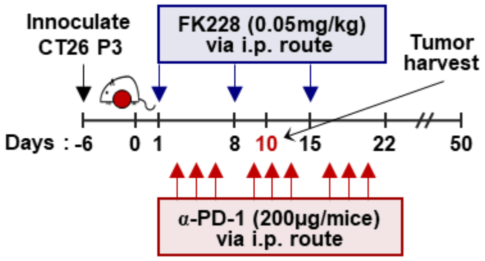

Supplemental Figure 17. Diagram depicting the schedule of *in vivo* treatment. Schematic of the therapy regimen in BALB/c mice implanted with CT26 P3 cells.

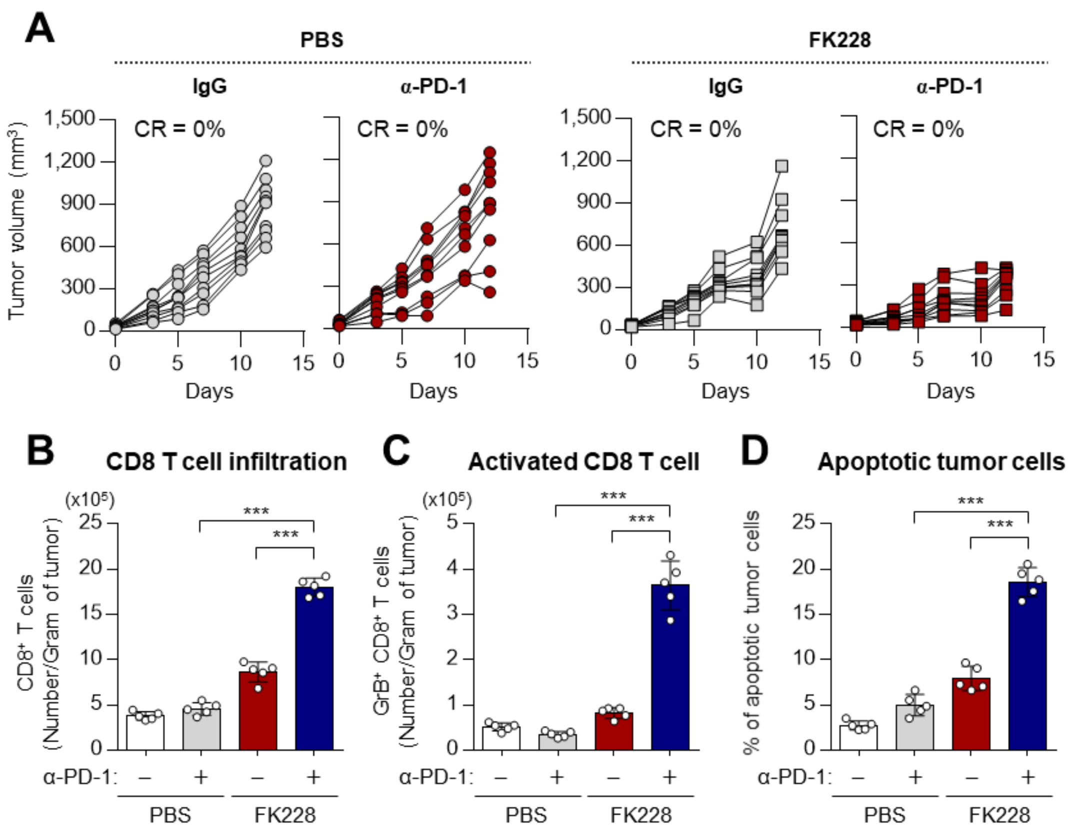

Supplemental Figure 18. Targeting HDAC1 overcomes resistance to anti-PD-1 therapy in YUMM2.1 P3. (**A** to **D**) YUMM2.1 P3 tumor-bearing mice administrated vehicle of FK228, with or without PD-1 antibody. (**A**) Tumor growth curves. (**B**) Flow cytometry profiles of tumor-infiltrating $CD3^+$ $CD8^+$ T cells. (**C**) The ratio of granzyme B^+ to tumor-infiltrating $CD3^+$ $CD8^+$ T cells. (**D**) The frequency of apoptotic cells in the tumors. Ten mice from each group were used for in vivo experiments. The graphs represent three independent experiments performed in triplicate. (**B** to **D**) The p-values by one-way ANOVA are indicated. The data represent the mean ± SD. Source data are provided as a Source Data file. (*P ≤ 0.05, **P ≤ 0.01, ***P ≤ 0.001. NS, not significant)

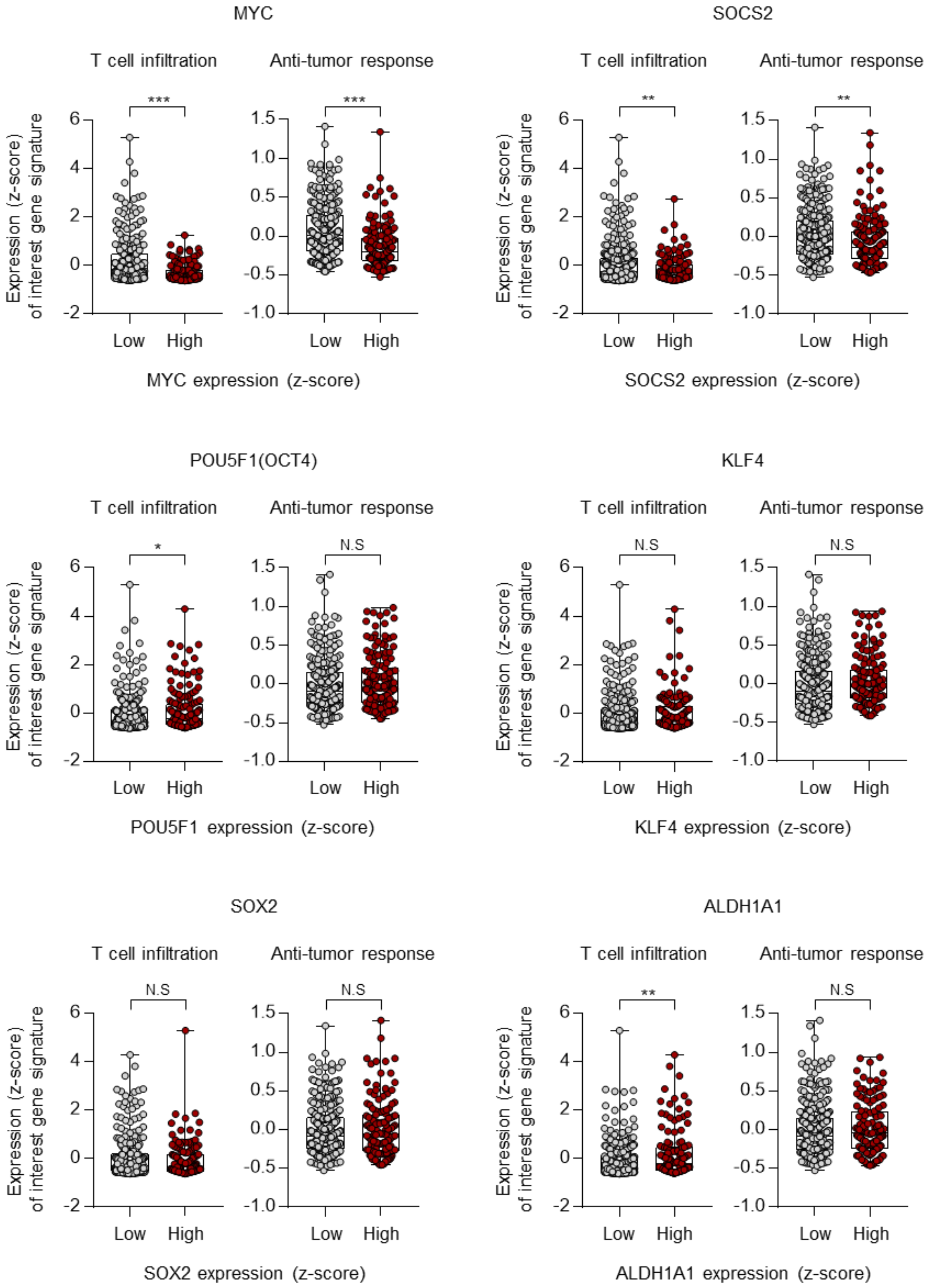

Supplemental Figure 19. MYC and SOCS2 are inversely associated with immune-refractory feature of the TME in melanoma patients. Comparisons of expression levels of T cell infiltration and anti-tumor response signatures in low levels and high levels of indicated genes. The 25th and 75th percentiles were used as cutoff thresholds. The p-values were determined by unpaired, two-tailed

Student's t-test. In the box plots, the top and bottom edges of boxes indicate the first and third quartiles, respectively; the center lines indicate the medians; and the ends of the whiskers indicate the maximum and minimum values, respectively. Source data are provided as a Source Data file. (*$P \leq 0.05$, **$P \leq 0.01$, ***$P \leq 0.001$. NS, not significant)

Supplemental Table 1. Differentially expressed genes between NANOG^{high} and NANOG^{low} patients.

Gene	AveExpr	logFC	p valuse	adj. p value
FAT1|2195	4.302713349	1.209476824	1.16875E-23	2.35305E-19
PPP4R1|9989	1.602767249	1.068143905	1.89407E-12	1.90666E-08
ANKRD57|65124	1.839260911	1.109958	3.50392E-11	2.35148E-07
RNF217|154214	2.086034504	1.219251444	3.29202E-10	1.65696E-06
CSK|1445	0.619978689	0.94230173	1.37199E-09	5.2357E-06
VRK3|51231	0.755125851	0.95812483	1.56033E-09	5.2357E-06
PARD3|56288	1.683417381	1.08529568	2.18288E-09	6.27827E-06
PSD3|23362	2.896094047	1.190925024	2.78468E-09	6.93998E-06
RUNX3|864	0.477668685	0.907871606	3.44707E-09	6.93998E-06
SH3D19|152503	2.026149312	1.116206449	3.30644E-09	6.93998E-06
MANSC1|54682	2.28498751	1.157999227	4.4289E-09	8.10609E-06
RBCK1|10616	0.697182502	0.954702728	6.86646E-09	1.15202E-05
GAN|8139	1.961230094	1.189796425	7.91336E-09	1.22554E-05
MIIP|60672	0.64712949	0.935764836	9.64391E-09	1.38686E-05
TMCO4|255104	0.696694085	0.942458973	1.60798E-08	2.15823E-05
SPESP1|246777	12.6214698	1.759516239	1.742E-08	2.19198E-05
MAPK6|5597	1.609512208	1.071558852	1.86435E-08	2.20794E-05
USP46|64854	1.503928213	1.06913879	2.6829E-08	2.94643E-05
YWHAQ|10971	1.291643926	1.030174864	2.78061E-08	2.94643E-05
MARVELD2|153562	4.877985544	1.730404013	3.15491E-08	3.17589E-05
TMEM143|55260	0.626986273	0.912964048	3.53988E-08	3.39374E-05
HMHA1|23526	0.432348063	0.889473649	3.87775E-08	3.54867E-05
KLF10|7071	1.612144582	1.072241499	4.91797E-08	4.30493E-05
PLEKHO2|80301	0.650805029	0.949099923	5.68928E-08	4.7726E-05
TET2|54790	1.661778707	1.090198156	5.95035E-08	4.79194E-05
AIP|9049	0.646405609	0.94334091	6.295E-08	4.87451E-05
TUSC3|7991	2.036648031	1.109139146	6.68152E-08	4.98218E-05
FBXO44|93611	0.649716104	0.935526802	7.1894E-08	5.16943E-05
TMTC3|160418	1.600504797	1.081958719	7.87601E-08	5.46785E-05
FBXO6|26270	0.552971482	0.906977419	8.36916E-08	5.4721E-05
PERP|64065	8.197207725	1.305672876	8.42573E-08	5.4721E-05
KLF5|688	4.957578933	1.337303516	8.78582E-08	5.47515E-05
SH2B2|10603	0.485831073	0.847156019	8.97432E-08	5.47515E-05
ZNF778|197320	1.500334971	1.095267286	9.62621E-08	5.70013E-05
LCP2|3937	0.491033225	0.898207033	1.04573E-07	5.93101E-05
PTPN18|26469	0.642515042	0.941973429	1.06053E-07	5.93101E-05
C19orf66|55337	0.644451029	0.937719111	1.16272E-07	6.26959E-05
LIMD2|80774	0.485211506	0.90781405	1.18335E-07	6.26959E-05
CRTC2|200186	0.69894671	0.951721084	1.22384E-07	6.31786E-05
BICD1|636	1.595567145	1.086314983	1.44196E-07	6.6508E-05
EXPH5|23086	6.220536822	1.708198989	1.45351E-07	6.6508E-05
ICAM3|3385	0.422044113	0.874910018	1.38426E-07	6.6508E-05
NCF1|653361	0.244675332	0.778820709	1.34458E-07	6.6508E-05
RHBDL2|54933	5.90609982	1.962915511	1.42724E-07	6.6508E-05
LSM10|84967	0.746230354	0.957250965	1.57209E-07	6.93503E-05
MPND|84954	0.617930654	0.926167298	1.6491E-07	6.93503E-05
RAB20|55647	0.437325967	0.871801412	1.65341E-07	6.93503E-05

SLC20A2|6575	1.568307783	1.069635052	1.60899E-07	6.93503E-05
APBA3|9546	0.701369446	0.941807732	1.75028E-07	7.04887E-05
MBOAT2|129642	1.702176647	1.092295958	1.75058E-07	7.04887E-05
NCF1C|654817	0.22611928	0.720694429	1.83463E-07	7.24247E-05
UNC93B1|81622	0.543956423	0.913565279	1.89615E-07	7.34137E-05
HSPA4L|22824	2.268074005	1.170183418	2.08201E-07	7.90888E-05
FUCA1|2517	0.517209623	0.911239982	2.16093E-07	8.05665E-05
GPR115|221393	80.24701011	-27.25654475	2.46958E-07	9.03999E-05
C12orf29|91298	1.446469913	1.06797829	2.7243E-07	9.14139E-05
DSP|1832	26.49566193	1.54553027	2.62484E-07	9.14139E-05
GMIP|51291	0.562006919	0.914811352	2.70329E-07	9.14139E-05
OCEL1|79629	0.589305332	0.910687701	2.64449E-07	9.14139E-05
STAT5A|6776	0.648549015	0.944912841	2.58372E-07	9.14139E-05
PYCR2|29920	0.729754103	0.959759218	2.78267E-07	9.18417E-05
AKR1A1|10327	0.66623391	0.948650097	3.03565E-07	9.78017E-05
PVRIG|79037	0.321295302	0.789737084	3.06978E-07	9.78017E-05
VSNL1|7447	25.85150985	3.044958856	3.10898E-07	9.78017E-05
EBI3|10148	0.408236742	0.816212144	3.21715E-07	9.81378E-05
VTA1|51534	1.374586491	1.048573209	3.17984E-07	9.81378E-05
ARHGEF1|9138	0.667773397	0.950867811	3.39104E-07	0.0001004
HCST|10870	0.359292465	0.834270241	3.37042E-07	0.0001004
BZW1|9689	1.369052669	1.040016116	3.636E-07	0.000102319
GSTM4|2948	0.57586133	0.923963281	3.68633E-07	0.000102319
LOC728743|728743	0.593422018	0.909443263	3.70996E-07	0.000102319
SEPT10|151011	1.452729789	1.055447681	3.66466E-07	0.000102319
UBE2E3|10477	1.479998231	1.056796771	3.6537E-07	0.000102319
APBB2|323	1.831152343	1.098868162	3.85814E-07	0.000104289
KCTD10|83892	1.263167547	1.032409082	3.88501E-07	0.000104289
HDAC2|3066	1.43430083	1.049476605	3.93941E-07	0.000104358
TRAF2|7186	0.697650628	0.945921679	4.16173E-07	0.000108816
APPL2|55198	1.442226188	1.05566722	4.34454E-07	0.000112139
ANKRD58|347454	0.330695096	0.726174818	4.7375E-07	0.000114427
DENND4B|9909	0.745250109	0.961464822	4.69396E-07	0.000114427
IRF3|3661	0.718694557	0.954425099	4.7742E-07	0.000114427
LRCH4|4034	0.696593966	0.953010329	4.72645E-07	0.000114427
TMEM30A|55754	1.529076525	1.054753748	4.76035E-07	0.000114427
TNFSF12|8742	0.552967068	0.916014317	4.52747E-07	0.000114427
DCUN1D3|123879	1.469231408	1.079802518	5.07678E-07	0.000120248
PTPN13|5783	1.932079055	1.101333151	5.17367E-07	0.000121118
ANGPTL6|83854	0.390714574	0.720984297	5.3146E-07	0.000121136
ASCC3|10973	1.501167155	1.061891736	5.28088E-07	0.000121136
C17orf87|388325	0.330000007	0.739559632	5.35493E-07	0.000121136
HECTD1|25831	1.4308548	1.046953103	5.45127E-07	0.000121945
TPBG|7162	2.631352064	1.19283467	5.61018E-07	0.000124121
DDIT4L|115265	8.997426738	1.635747748	6.07732E-07	0.000132994
IPPK|64768	1.609686833	1.08488	6.19743E-07	0.000134164
C19orf54|284325	0.744039046	0.95256023	6.75909E-07	0.000142529
COL17A1|1308	76.46654656	2.290902104	6.87747E-07	0.000142529
PNKD|25953	0.673450707	0.949121998	6.74304E-07	0.000142529
TMEM149|79713	0.441559267	0.869509161	6.9378E-07	0.000142529
ZNF672|79894	0.692811297	0.949949411	6.92592E-07	0.000142529
HIGD2A|192286	0.569989355	0.927528526	7.04238E-07	0.000143216

SIRPG|55423	0.291380236	0.776793043	7.1408E-07	0.000143766
SH3RF2|153769	18.91025503	2.268822545	7.23657E-07	0.000144251
SERPINB8|5271	1.920872933	1.117508384	7.31192E-07	0.000144324
PSMB9|5698	0.478101906	0.900430043	7.53445E-07	0.000145029
RASSF9|9182	3.506273677	1.771545718	7.56374E-07	0.000145029
WAS|7454	0.367132979	0.846443341	7.50617E-07	0.000145029
SIGLEC5|8778	0.376552504	0.767093648	7.65416E-07	0.000145378
EXOC1|55763	1.335093878	1.044464029	7.81981E-07	0.000146403
TAPBP|6892	0.614831993	0.947427814	7.90468E-07	0.000146403
TFEB|7942	0.535736368	0.903567657	7.92625E-07	0.000146403
TIFAB|497189	0.340387675	0.718434792	8.09877E-07	0.00014823
ABHD14B|84836	0.616023377	0.939440741	8.71172E-07	0.000149909
BST2|684	0.562152169	0.931975965	8.40299E-07	0.000149909
CSDA|8531	1.551691783	1.055892552	8.70911E-07	0.000149909
ING2|3622	1.364495899	1.063008684	8.57E-07	0.000149909
METAP1|23173	1.316328571	1.0414956	8.53622E-07	0.000149909
NFKB2|4791	0.639412017	0.940361511	8.64044E-07	0.000149909
SH3BGRL2|83699	2.06366049	1.159359168	8.48479E-07	0.000149909
CNPY3|10695	0.6739549	0.950655213	8.83421E-07	0.000150167
IL20RB|53833	18.43079111	2.122950449	9.02509E-07	0.000150167
KLF8|11279	2.426993472	1.241582576	8.89097E-07	0.000150167
ZNF229|7772	2.660953245	1.229045069	8.9918E-07	0.000150167
BLVRB|645	0.603906323	0.931561142	9.55169E-07	0.000151857
CD7|924	0.286612591	0.81030399	9.39227E-07	0.000151857
CDS1|1040	4.414458131	1.467292105	9.23003E-07	0.000151857
KLHDC5|57542	1.368535176	1.047914938	9.29417E-07	0.000151857
NADK|65220	0.785825249	0.967327876	9.47155E-07	0.000151857
RAB27B|5874	12.23835624	20.79996305	9.5792E-07	0.000151857
ARL5A|26225	1.362275931	1.048100881	9.83635E-07	0.000152396
C1orf106|55765	6.386659596	1.485261474	9.74375E-07	0.000152396
IMPAD1|54928	1.479411479	1.050792392	9.84029E-07	0.000152396
AATF|26574	0.760552861	0.964530578	1.04274E-06	0.000154363
FAM160A1|729830	5.414637255	1.754129283	1.01327E-06	0.000154363
METAP2|10988	1.332946854	1.039527652	1.03801E-06	0.000154363
RAB4B|53916	0.638982295	0.933778855	1.03103E-06	0.000154363
SPTLC3|55304	3.663457171	1.33809674	1.0414E-06	0.000154363
ZBTB48|3104	0.756463987	0.954051275	1.04215E-06	0.000154363
GALM|130589	0.584806072	0.918368292	1.12822E-06	0.000155135
GFOD2|81577	1.32077276	1.044578667	1.08312E-06	0.000155135
GNGT2|2793	0.428722696	0.780746238	1.12614E-06	0.000155135
LRRC1|55227	1.815822594	1.108935489	1.13119E-06	0.000155135
NDFIP2|54602	1.469063189	1.059489152	1.07888E-06	0.000155135
PANK4|55229	0.76719012	0.959909146	1.11696E-06	0.000155135
PAWR|5074	1.905999181	1.160788262	1.13271E-06	0.000155135
PPP2CB|5516	1.346434011	1.041227114	1.09929E-06	0.000155135
PRDM4|11108	1.27515246	1.037012066	1.12022E-06	0.000155135
SDR42E1|93517	5.581728952	1.758980873	1.12512E-06	0.000155135
ZNF662|389114	2.430281927	1.226203096	1.09539E-06	0.000155135
TAZ|6901	0.731551799	0.9509504	1.14497E-06	0.000155755
EGLN2|112398	0.771028985	0.966559241	1.17007E-06	0.000157204
MTMR14|64419	0.764440012	0.962692031	1.17124E-06	0.000157204
ITGAL|3683	0.310942745	0.841765717	1.1913E-06	0.000157792

KIF5B|3799	1.44564616	1.047260831	1.18752E-06	0.000157792
CD72|971	0.35260373	0.820498548	1.20483E-06	0.000158541
GJA1|2697	5.431133635	1.270996062	1.22227E-06	0.000158761
TMEM184C|55751	1.312281922	1.043638429	1.22082E-06	0.000158761
ATMIN|23300	1.283857515	1.035590209	1.25639E-06	0.000159867
IL2RG|3561	0.3198545	0.846780811	1.26828E-06	0.000159867
PPM1M|132160	0.673010697	0.940160179	1.27049E-06	0.000159867
PTPRCAP|5790	0.265411989	0.811738538	1.26209E-06	0.000159867
ZDHHC18|84243	0.732279607	0.9554557	1.24763E-06	0.000159867
C1orf66|51093	0.711723068	0.946782051	1.28888E-06	0.000160179
MAD1L1|8379	0.46703115	0.905278236	1.28865E-06	0.000160179
CXADR|1525	4.230301902	1.417671305	1.33018E-06	0.000164298
SLC25A19|60386	0.708836024	0.943149907	1.3438E-06	0.000164968
PTPN6|5777	0.356246992	0.86013367	1.3596E-06	0.000165895
PDLIM5|10611	1.489102239	1.057855741	1.42022E-06	0.000172249
KCNK1|3775	8.657766586	2.138599865	1.45707E-06	0.00017566
ITGB7|3695	0.358854375	0.841754026	1.4695E-06	0.000175957
SYNCRIP|10492	1.291168851	1.032330887	1.47701E-06	0.000175957
MXD1|4084	1.692892487	1.097327327	1.48866E-06	0.000175987
PRKCD|5580	0.759438536	0.964935218	1.49475E-06	0.000175987
MAST3|23031	0.681948186	0.941343975	1.51693E-06	0.00017756
DCPS|28960	0.694948018	0.947205554	1.55186E-06	0.000180571
PDGFC|56034	3.290632744	1.257095427	1.56059E-06	0.000180571
ADAM9|8754	1.951591	1.096848935	1.57217E-06	0.00018069
CD300LF|146722	0.378506879	0.795201646	1.57957E-06	0.00018069
FAM78A|286336	0.569747814	0.916473931	1.61401E-06	0.000183587
C1QB|713	0.367898721	0.891261382	1.63602E-06	0.000185044
CD27|939	0.26537638	0.803443239	1.64864E-06	0.00018543
EPB41L4B|54566	4.735582354	1.578218179	1.68246E-06	0.000188183
EPOR|2057	0.644351372	0.925021967	1.69969E-06	0.000189059
SAFB2|9667	0.827692361	0.974729559	1.73006E-06	0.000189697
SLA|6503	0.433366146	0.874192114	1.72658E-06	0.000189697
THAP8|199745	0.694893784	0.934016135	1.73368E-06	0.000189697
BRI3|25798	0.679948196	0.953323929	1.78036E-06	0.000193751
NCF1B|654816	0.248424046	0.716843747	1.83483E-06	0.000198606
ATP8B1|5205	2.162482826	1.176154575	1.94454E-06	0.000200766
C17orf62|79415	0.702510155	0.953513646	1.91597E-06	0.000200766
CHMP4C|92421	6.738710222	1.938042605	1.91014E-06	0.000200766
DENND1C|79958	0.333304157	0.825114724	1.93748E-06	0.000200766
DENND2C|163259	2.312198619	1.25233558	1.87225E-06	0.000200766
FCER1G|2207	0.288956117	0.839387539	1.93399E-06	0.000200766
FRMD6|122786	1.68986971	1.073417587	1.92884E-06	0.000200766
SIPA1|6494	0.672360115	0.947924625	1.94127E-06	0.000200766
UCP2|7351	0.387957623	0.882416022	1.88597E-06	0.000200766
C1QA|712	0.372207575	0.891460592	2.02581E-06	0.00020639
GNRHR|2798	1.919065136	1.993346054	2.02977E-06	0.00020639
TACC2|10579	2.158862273	1.135848784	2.01349E-06	0.00020639
AKR1C2|1646	7.350351503	1.761739721	2.07103E-06	0.000209528
HLA-H|3136	0.537933296	0.928704006	2.08482E-06	0.000209868
RARS2|57038	1.404867236	1.056284508	2.1287E-06	0.000212164
ZNF185|7739	5.002869373	1.306539901	2.12205E-06	0.000212164
CWH43|80157	86.50543572	10.10347178	2.15064E-06	0.000213294

C1orf38|9473	0.562830262	0.918530059	2.16354E-06	0.000213523
C1QC|714	0.40023533	0.898270405	2.25217E-06	0.000219312
ECH1|1891	0.697477706	0.956381189	2.24708E-06	0.000219312
LNX1|84708	1.890051096	1.171744363	2.25488E-06	0.000219312
ANKRD56|345079	18.41167079	-5.385139132	2.28412E-06	0.000221088
CDK11B|984	0.780499017	0.965095909	2.33536E-06	0.00022125
EPT1|85465	1.370551651	1.04731549	2.31154E-06	0.00022125
GPR125|166647	1.43496614	1.053582157	2.33037E-06	0.00022125
HK3|3101	0.410550669	0.841068818	2.32318E-06	0.00022125
HLA-A|3105	0.556502086	0.947469086	2.34075E-06	0.00022125
C18orf8|29919	0.803569178	0.965351955	2.41899E-06	0.000224431
EPHB3|2049	2.239648526	1.133874151	2.40801E-06	0.000224431
MYO6|4646	1.839382185	1.103712712	2.39766E-06	0.000224431
P2RY11|5032	0.615606983	0.914410404	2.40938E-06	0.000224431
CAMK2D|817	1.616312728	1.072753093	2.46406E-06	0.000227037
DSG1|1828	139.2380553	2.778618085	2.46963E-06	0.000227037
GRIP1|23426	3.688798416	1.3617959	2.4882E-06	0.000227704
BCL2L2|599	1.358089685	1.04523099	2.5096E-06	0.000228142
IL12RB1|3594	0.396803696	0.833508911	2.5266E-06	0.000228142
SDR16C5|195814	43.98762773	3.877830643	2.52698E-06	0.000228142
PHF1|5252	0.749207649	0.961668951	2.54036E-06	0.000228326
ARID5A|10865	0.634504068	0.93386114	2.62833E-06	0.000233187
BICD2|23299	1.481758555	1.056201048	2.63079E-06	0.000233187
TMED10P1|286102	1.351778128	1.079544758	2.61707E-06	0.000233187
XRCC1|7515	0.779014137	0.964862167	2.64077E-06	0.000233187
NIPAL1|152519	3.409375838	1.645736208	2.65546E-06	0.00023346
ANXA8L2|244	74.32767933	3.944547846	2.67682E-06	0.000234315
ARFGEF1|10565	1.382036438	1.046829785	2.80595E-06	0.000238049
DENND2D|79961	0.394025914	0.861530634	2.84783E-06	0.000238049
ERI2|112479	1.337942087	1.055906271	2.78947E-06	0.000238049
JAG1|182	2.137064738	1.110782642	2.79525E-06	0.000238049
KIAA1919|91749	1.426555084	1.085145895	2.7572E-06	0.000238049
LYL1|4066	0.468365572	0.861408537	2.86013E-06	0.000238049
MRM1|79922	0.718434503	0.94059495	2.82821E-06	0.000238049
SERPINB5|5268	154.7186892	3.568106624	2.78705E-06	0.000238049
SMAD1|4086	1.862904488	1.118918039	2.73134E-06	0.000238049
TREX1|11277	0.61624545	0.924735029	2.87318E-06	0.000238049
UBA6|55236	1.419288515	1.053958631	2.75323E-06	0.000238049
VPS16|64601	0.751274658	0.961129346	2.8489E-06	0.000238049
ZNF268|10795	1.495284587	1.074897548	2.87118E-06	0.000238049
ACBD4|79777	0.670859184	0.928154157	2.90901E-06	0.000240029
PSMB10|5699	0.580322778	0.929278053	2.94007E-06	0.000241602
PAFAH1B1|5048	1.250550056	1.028444799	2.99756E-06	0.000244028
S100A16|140576	3.161064988	1.152966123	3.00596E-06	0.000244028
TMEM150A|129303	0.734600285	0.950643544	2.98738E-06	0.000244028
FAM110C|642273	35.9637957	-1.629393377	3.12053E-06	0.000252102
MPP7|143098	5.146669173	1.576425856	3.13045E-06	0.000252102
TSPYL4|23270	1.427816841	1.058498293	3.14493E-06	0.000252258
CUTA|51596	0.642453143	0.948508729	3.16165E-06	0.000252396
PVRL4|81607	15.21239755	1.888388255	3.20015E-06	0.000252396
SLA2|84174	0.353730949	0.798600832	3.20933E-06	0.000252396
THAP3|90326	0.684441473	0.938930192	3.17999E-06	0.000252396

UBXN11|91544	0.678587754	0.938793484	3.20025E-06	0.000252396
ABI3|51225	0.42805462	0.861972687	3.29372E-06	0.000255184
PRR13|54458	0.761658586	0.965012206	3.29548E-06	0.000255184
RILPL2|196383	0.656619301	0.928485269	3.26575E-06	0.000255184
STK38L|23012	1.519687734	1.069639974	3.28224E-06	0.000255184
DNAJC5B|85479	0.311650868	0.666074161	3.33252E-06	0.000256083
FAM168B|130074	1.254509807	1.028566785	3.3226E-06	0.000256083
FAM83B|222584	94.10363639	-11.3375574	3.36067E-06	0.000257264
SMARCAD1|56916	1.575392977	1.069231783	3.38582E-06	0.000258207
RAET1E|135250	24.61385544	5.559032648	3.42636E-06	0.000259334
TOR2A|27433	0.662243037	0.931597969	3.41686E-06	0.000259334
MYO1G|64005	0.382895806	0.84791176	3.46654E-06	0.000261392
MAST4|375449	2.311648836	1.17786031	3.49698E-06	0.000262704
C19orf38|255809	0.365417397	0.767610789	3.53403E-06	0.000263963
MAD2L2|10459	0.686356588	0.947833252	3.55804E-06	0.000263963
TMEM181|57583	1.486773402	1.058492577	3.54245E-06	0.000263963
TPRA1|131601	0.685769789	0.946409195	3.56619E-06	0.000263963
S1PR4|8698	0.31969173	0.782214942	3.63483E-06	0.000268058
TRAPPC6A|79090	0.59330789	0.917946686	3.68823E-06	0.000271004
ANXA8|653145	59.0348989	2.644088244	3.71742E-06	0.000272155
TNKS1BP1|85456	1.603557286	1.061254698	3.77794E-06	0.000275584
CTSH|1512	0.582039335	0.934803329	3.855E-06	0.000280191
SIGLEC14|100049587	0.398053689	0.805815316	3.88757E-06	0.000281541
STAT5B|6777	0.781446319	0.967520161	3.98248E-06	0.000287381
VEZT|55591	1.351069889	1.048030059	4.02326E-06	0.000289287
CHD9|80205	1.456036545	1.056343662	4.04337E-06	0.000289485
PITPNM3|83394	3.701431911	1.380229051	4.05478E-06	0.000289485
IDE|3416	1.634480565	1.085280119	4.09452E-06	0.000290827
SEC24B|10427	1.336939105	1.043600853	4.10701E-06	0.000290827
SLAMF8|56833	0.430360803	0.879562676	4.13843E-06	0.000290827
SOX7|83595	3.206063236	1.266424996	4.12585E-06	0.000290827
ZNF770|54989	1.446683557	1.057260055	4.1458E-06	0.000290827
CORO1A|11151	0.318552917	0.861632912	4.17955E-06	0.000292177
MAL2|114569	13.56716048	1.757965114	4.2232E-06	0.000293192
SIT1|27240	0.265799935	0.758609768	4.21062E-06	0.000293192
PLA2G2D|26279	0.163832438	0.734432755	4.34623E-06	0.000300696
PSMF1|9491	0.793856933	0.972119759	4.36962E-06	0.000301279
AKNA|80709	0.440332598	0.890044021	4.49862E-06	0.000303364
CAPNS2|84290	62.77582077	5.680991107	4.44465E-06	0.000303364
CCDC22|28952	0.741197356	0.955493911	4.5204E-06	0.000303364
CKAP4|10970	1.523476431	1.054439111	4.47713E-06	0.000303364
DHX32|55760	1.298602203	1.040942033	4.50847E-06	0.000303364
RNF130|55819	0.748282136	0.962109017	4.43469E-06	0.000303364
SHISA5|51246	0.758540882	0.968046234	4.46846E-06	0.000303364
STAU2|27067	1.347601931	1.047964462	4.45135E-06	0.000303364
GSTK1|373156	0.693712505	0.955750186	4.54067E-06	0.000303712
CXCR2|3579	5.122122229	1.852309053	4.62046E-06	0.000305999
LRRC23|10233	0.707451173	0.937963252	4.61812E-06	0.000305999
NCKAP1|10787	1.358690028	1.038601195	4.6003E-06	0.000305999
HS1BP3|64342	0.703976614	0.951685601	4.69548E-06	0.000308935
RHEBL1|121268	0.678108003	0.91469885	4.69303E-06	0.000308935
DHX58|79132	0.632368989	0.926896786	4.80126E-06	0.000314865

CD40|958	0.488973273	0.887751587	4.86071E-06	0.000317133
FMNL1|752	0.438891362	0.884998312	4.86734E-06	0.000317133
TACSTD2|4070	28.91276437	1.880673565	4.91477E-06	0.000319191
CD247|919	0.323740105	0.803619546	5.00862E-06	0.00032424
ATP6V1F|9296	0.621028667	0.944643481	5.09049E-06	0.000328484
DENR|8562	1.282119023	1.035236031	5.12894E-06	0.000328772
EDEM2|55741	0.768479095	0.962507502	5.20927E-06	0.000328772
NCKAP1L|3071	0.442024887	0.882328047	5.20336E-06	0.000328772
SLAMF6|114836	0.311492803	0.802560426	5.18705E-06	0.000328772
TRIM6|117854	1.912499148	1.140458162	5.17566E-06	0.000328772
TYK2|7297	0.788437925	0.968839419	5.20032E-06	0.000328772
ZNF323|64288	1.690385042	1.129480675	5.14728E-06	0.000328772
CDK2AP2|10263	0.575272088	0.926319794	5.29722E-06	0.000332245
GRHL1|29841	12.81402732	1.83511991	5.29731E-06	0.000332245
ICAM1|3383	0.602126675	0.942594762	5.32777E-06	0.000333118
RANGRF|29098	0.72525397	0.951367124	5.38912E-06	0.000335911
TSPAN33|340348	0.608480945	0.925278305	5.4537E-06	0.000338887
MAP4K5|11183	1.469435622	1.059901775	5.51622E-06	0.000341717
PTPN11|5781	1.330802625	1.037053396	5.55966E-06	0.000343352
SEPT1|1731	0.289172116	0.805886687	5.62461E-06	0.0003463
MICALCL|84953	8.219641344	3.072941746	5.64691E-06	0.000346614
CD53|963	0.392476879	0.87472763	5.72851E-06	0.000350554
ARHGAP32|9743	1.627103748	1.076576175	5.78044E-06	0.000352659
CAPZB|832	0.79055907	0.973097504	5.82508E-06	0.000352716
HAX1|10456	0.730548973	0.960036594	5.82553E-06	0.000352716
LAPTM5|7805	0.460035248	0.912830962	5.83393E-06	0.000352716
LCK|3932	0.314320961	0.823229615	5.8788E-06	0.000354365
LRP6|4040	1.503275638	1.060681199	5.94567E-06	0.000355205
MAP3K7|6885	1.480864565	1.063284986	5.9436E-06	0.000355205
VAV1|7409	0.384469417	0.844373269	5.92081E-06	0.000355205
TCF7L2|6934	1.367314916	1.053188583	6.01079E-06	0.000358033
PDCD1|5133	0.276280104	0.77494491	6.05707E-06	0.000359726
MAP4K1|11184	0.410616959	0.860505249	6.14291E-06	0.00036144
SCEL|8796	18.80432336	2.280674677	6.14089E-06	0.00036144
TMEM184A|202915	26.83444839	2.788566699	6.13441E-06	0.00036144
ZNF750|79755	69.95448293	3.687260012	6.15774E-06	0.00036144
PYGO2|90780	0.75111863	0.962229302	6.2263E-06	0.000364401
SLC10A6|345274	8.122665014	3.703849664	6.30475E-06	0.000367923
POLR3B|55703	1.298503023	1.047705709	6.33759E-06	0.000368771
DNPEP|23549	0.772873775	0.965349752	6.36548E-06	0.000369326
MAN2B1|4125	0.643775586	0.950221677	6.46838E-06	0.000372717
RBM10|8241	0.822858156	0.97453595	6.48566E-06	0.000372717
SETD8|387893	1.233136737	1.032141198	6.49797E-06	0.000372717
TBX21|30009	0.356040181	0.770558061	6.45357E-06	0.000372717
GRK6|2870	0.644146758	0.941257033	6.54136E-06	0.00037414
SMAGP|57228	3.503940244	1.308199413	6.6033E-06	0.000376613
DNASE2|1777	0.725484403	0.958531967	6.68828E-06	0.000380381
CD6|923	0.34756437	0.830717213	6.82138E-06	0.000386859
ERLIN2|11160	1.394261667	1.047741921	6.8633E-06	0.000387056
RPL23AP53|644128	1.454315207	1.073477709	6.85507E-06	0.000387056
TRPM6|140803	3.688031213	1.518212317	6.98064E-06	0.000392573
IL1F5|26525	98.9339957	5.037025266	7.00325E-06	0.00039268

MUTYH|4595	0.721639746	0.944044975	7.02155E-06	0.00039268
RAVER1|125950	0.741099975	0.962133983	7.04476E-06	0.000392887
FAM179B|23116	1.394845159	1.05808553	7.06776E-06	0.000393081
ANKRD50|57182	1.613387299	1.070132901	7.18726E-06	0.000396441
GPR87|53836	113.5654337	-16.56996357	7.15442E-06	0.000396441
TPM1|7168	1.706001187	1.077006926	7.18315E-06	0.000396441
IFI35|3430	0.588391363	0.927305212	7.20794E-06	0.000396496
CLCA2|9635	101.4486133	2.817164811	7.27261E-06	0.000397879
VAMP5|10791	0.555670211	0.919252041	7.27078E-06	0.000397879
BATF|10538	0.521340045	0.885278375	7.31358E-06	0.000399036
SEC63|11231	1.323287814	1.037463713	7.34042E-06	0.000399418
IL22RA1|58985	7.68845211	1.72042331	7.42607E-06	0.000402989
SGCB|6443	1.606328499	1.072988688	7.48118E-06	0.000404889
BNIP3L|665	1.372422117	1.042077844	7.58898E-06	0.000407205
DSC3|1825	118.7620918	2.672145448	7.56194E-06	0.000407205
SPOCK2|9806	0.364633872	0.863531865	7.57709E-06	0.000407205
TP53I13|90313	0.647566898	0.938462444	7.60488E-06	0.000407205
AEBP2|121536	1.342020468	1.047211669	7.75428E-06	0.000413801
GHDC|84514	0.682449176	0.947597333	7.81028E-06	0.000413801
SASH3|54440	0.327993688	0.84004355	7.80628E-06	0.000413801
SPN|6693	0.405274704	0.857198238	7.78953E-06	0.000413801
PEX14|5195	0.786231324	0.962844813	7.85706E-06	0.000415187
BNIPL|149428	46.45736988	3.212140184	7.88458E-06	0.00041555
PCBP1|5093	0.84297704	0.981416657	8.03273E-06	0.000422253
AKR7A2|8574	0.713103794	0.954011719	8.17046E-06	0.000425192
ARHGAP9|64333	0.336490552	0.827956892	8.20543E-06	0.000425192
CSDAP1|440359	1.839755176	1.185222302	8.12065E-06	0.000425192
DIO2|1734	4.835236414	1.354070797	8.17782E-06	0.000425192
ID1|3397	3.68690514	1.260219971	8.20955E-06	0.000425192
SRM|6723	0.685729589	0.955743617	8.21536E-06	0.000425192
QSER1|79832	1.407455791	1.050857674	8.24297E-06	0.000425528
CD3E|916	0.308715312	0.836783202	8.32467E-06	0.000427567
FERMT3|83706	0.517064441	0.911841775	8.32496E-06	0.000427567
NCF4|4689	0.49938998	0.883716442	8.43459E-06	0.000432096
DOK3|79930	0.477357002	0.88207006	8.50809E-06	0.000432777
FAM13A|10144	1.632796552	1.08509391	8.63352E-06	0.000432777
GSTCD|79807	1.410758836	1.07010402	8.59557E-06	0.000432777
HLA-DMB|3109	0.468139961	0.900366432	8.59967E-06	0.000432777
KIAA1217|56243	2.068815841	1.123550993	8.57523E-06	0.000432777
LST1|7940	0.501324886	0.878523281	8.58324E-06	0.000432777
RPS6KA1|6195	0.632648533	0.937533275	8.58537E-06	0.000432777
SNAI3|333929	0.425773922	0.778981864	8.64135E-06	0.000432777
TRIAP1|51499	1.278318784	1.039785913	8.50832E-06	0.000432777
DOCK5|80005	1.987540369	1.130998549	8.69256E-06	0.000434261
IRF5|3663	0.525960647	0.893069916	8.77766E-06	0.000437427
GRB7|2886	13.84916501	2.623491724	8.84196E-06	0.00043943
PPL|5493	8.512909758	1.380317057	8.86151E-06	0.00043943
ROBLD3|28956	0.635453149	0.937629482	8.92163E-06	0.000441325
C4orf36|132989	1.874433439	1.839800447	9.01957E-06	0.00044446
TM9SF3|56889	1.293988601	1.032727251	9.02917E-06	0.00044446
NPR3|4883	7.029702151	3.023627465	9.18453E-06	0.000451005
AADAT|51166	1.837441586	1.141104398	9.23451E-06	0.000452356

PPP2R2C|5522	15.31817306	1.964909621	9.42117E-06	0.00046038
RASSF10|644943	5.053481153	2.159856709	9.46374E-06	0.00046134
ST3GAL3|6487	0.733217993	0.949918388	9.49608E-06	0.000461798
EIF4E|1977	1.294339294	1.043714878	9.54755E-06	0.000463183
WDFY3|23001	1.381981048	1.049299322	9.57291E-06	0.000463297
TMEM87A|25963	1.344089754	1.04061952	9.60504E-06	0.000463737
SOX13|9580	0.651663515	0.946724611	9.63893E-06	0.00046426
LGALS9|3965	0.489676436	0.906120635	9.67154E-06	0.000464719
LAGE3|8270	0.660455949	0.938817884	9.71701E-06	0.000465792
HCK|3055	0.518863688	0.898689544	9.81046E-06	0.000468899
IL4I1|259307	0.356808175	0.842914554	9.82841E-06	0.000468899
GIMAP5|55340	0.461162329	0.885196439	9.86544E-06	0.000469553
CYB561D2|11068	0.731559522	0.951782288	9.90799E-06	0.000470466
HMGCL|3155	0.776931896	0.963108823	9.98584E-06	0.000471936
LIMCH1|22998	2.70341755	1.188353467	9.98334E-06	0.000471936
FABP5|2171	4.589473624	1.279964569	1.00343E-05	0.000473115
GSDMC|56169	32.45263533	3.893154172	1.00581E-05	0.000473129
CHST12|55501	0.704480786	0.946175182	1.01911E-05	0.00047385
GLTP|51228	2.232883112	1.117590321	1.01881E-05	0.00047385
NUS1|116150	1.376350119	1.046988055	1.01289E-05	0.00047385
TIMM17B|10245	0.717882344	0.951623316	1.01427E-05	0.00047385
UBA7|7318	0.605285291	0.930021086	1.01646E-05	0.00047385
ARRB2|409	0.587511731	0.924821739	1.024E-05	0.000473935
PSMD7|5713	1.230140351	1.028551333	1.02281E-05	0.000473935
ARL8A|127829	0.748839033	0.961472239	1.03087E-05	0.000474996
CD3D|915	0.329905149	0.816715041	1.03347E-05	0.000474996
SLC2A6|11182	0.384229588	0.845290361	1.03573E-05	0.000474996
SPI1|6688	0.408249184	0.874030087	1.03477E-05	0.000474996
PTPRK|5796	1.723075204	1.091211112	1.04133E-05	0.000476478
ACVR2A|92	1.48271123	1.073634464	1.04526E-05	0.000477195
CCNC|892	1.434657454	1.057255702	1.05403E-05	0.000480108
NPRL2|10641	0.785032713	0.960505524	1.06091E-05	0.000482152
STRAP|11171	1.298218547	1.034064032	1.06702E-05	0.000483837
LOC606724|606724	0.351022118	0.776438032	1.0777E-05	0.000487579
PGM3|5238	1.498203836	1.072494097	1.08269E-05	0.000487646
SIGLEC10|89790	0.410214638	0.848057921	1.08146E-05	0.000487646
GTF2H3|2967	1.396502997	1.06492936	1.09008E-05	0.000489881
MACF1|23499	1.328712145	1.034244875	1.10102E-05	0.000493524
SYT8|90019	21.10041358	17.3068893	1.10309E-05	0.000493524
PTPRF|5792	1.974781272	1.093508331	1.10897E-05	0.000495051
SLC38A4|55089	3.817947944	3.436330958	1.11742E-05	0.000497723
ZBTB17|7709	0.77820804	0.962253498	1.12034E-05	0.000497922
CCDC6|8030	1.301186802	1.039764658	1.12621E-05	0.000498367
LRRC25|126364	0.417644494	0.85244187	1.12696E-05	0.000498367
MPZL2|10205	4.271644871	1.294043916	1.12877E-05	0.000498367
SH2D4A|63898	2.138668119	1.157141855	1.13198E-05	0.000498689
ITGB2|3689	0.395606368	0.890146985	1.13567E-05	0.000499222
EHF|26298	11.03805435	1.816628718	1.14917E-05	0.000499702
IL21R|50615	0.370980305	0.824007904	1.14763E-05	0.000499702
KCNJ15|3772	3.647762306	1.437981034	1.1426E-05	0.000499702
PLEK|5341	0.447501714	0.885236615	1.14514E-05	0.000499702
PSMB4|5692	0.757458526	0.969880541	1.14597E-05	0.000499702

ABHD12|26090	0.69463641	0.954424429	1.1567E-05	0.00049974
ATP13A2|23400	0.676333028	0.951463475	1.15519E-05	0.00049974
C7orf59|389541	0.712549073	0.956312845	1.15211E-05	0.00049974
ANO6|196527	1.382616045	1.045349006	1.15957E-05	0.000499905
BCL2L12|83596	0.732174037	0.949900525	1.1767E-05	0.000506209
C1orf161|126868	12.57656057	-9.355139414	1.18354E-05	0.000506411
GIMAP1|170575	0.482202954	0.863338499	1.18561E-05	0.000506411
SSR4|6748	0.644262723	0.949178667	1.18425E-05	0.000506411
USF2|7392	0.714308853	0.960234428	1.18723E-05	0.000506411
HOOK3|84376	1.362960226	1.045290065	1.19473E-05	0.000508531
FBXO45|200933	1.385728895	1.054216521	1.21291E-05	0.000513138
GJB3|2707	29.65696442	2.462145174	1.2132E-05	0.000513138
MAPKAPK5|8550	1.216777699	1.03258913	1.21076E-05	0.000513138
SLC47A2|146802	20.81414128	-255.1543409	1.21991E-05	0.000514892
AP1M1|8907	0.749100897	0.963574664	1.24526E-05	0.000523399
CCDC25|55246	1.331493634	1.04306674	1.24512E-05	0.000523399
MESP1|55897	0.536058477	0.842669843	1.25073E-05	0.000524603
ICAM2|3384	0.466093205	0.872829058	1.2591E-05	0.000525239
NIPAL2|79815	1.685947198	1.111700394	1.2551E-05	0.000525239
NR1H2|7376	0.710446055	0.956765247	1.26007E-05	0.000525239
CD68|968	0.468652325	0.916839046	1.26997E-05	0.00052827
ABCE1|6059	1.347230956	1.040742399	1.28253E-05	0.000529773
DSC1|1823	88.87792481	3.874447874	1.28674E-05	0.000529773
FABP5L3|220832	3.155730483	-10.74994951	1.27936E-05	0.000529773
NEDD4|4734	1.828551673	1.109006609	1.28626E-05	0.000529773
SENP6|26054	1.369447295	1.045785178	1.2804E-05	0.000529773
MEF2D|4209	0.705987459	0.956595981	1.29485E-05	0.000532025
MYO9B|4650	0.692745736	0.958124182	1.30498E-05	0.000532499
RWDD4A|201965	1.332508569	1.048765664	1.30658E-05	0.000532499
STRN3|29966	1.328141152	1.046853073	1.30234E-05	0.000532499
TNFAIP8L2|79626	0.460839523	0.851569127	1.30087E-05	0.000532499
TESK1|7016	0.688142874	0.946833077	1.32222E-05	0.000537782
RBM3|5935	1.314277794	1.032244581	1.32895E-05	0.00053943
ESYT3|83850	5.777701144	1.823588299	1.33709E-05	0.000541238
SMAP1|60682	1.28451039	1.04002024	1.33878E-05	0.000541238
NAGPA|51172	0.722494327	0.949506694	1.34242E-05	0.000541623
ABCA12|26154	85.648112	4.621526778	1.35092E-05	0.000542877
TMEM45A|55076	5.702114246	1.302257026	1.34845E-05	0.000542877
SYTL3|94120	0.50257933	0.86235595	1.36297E-05	0.000546628
ACVR1|90	1.40933324	1.051406291	1.3717E-05	0.000547415
ERGIC2|51290	1.361238673	1.049165185	1.37309E-05	0.000547415
PITPNB|23760	1.273520427	1.033370623	1.37268E-05	0.000547415
DSC2|1824	21.10447651	1.759147888	1.38959E-05	0.000552083
FGR|2268	0.394906806	0.84405133	1.39028E-05	0.000552083
RUFY1|80230	0.799752945	0.968261152	1.40204E-05	0.000554564
TRPV3|162514	4.236446607	1.753700309	1.4004E-05	0.000554564
RFXANK|8625	0.70340601	0.951417495	1.40502E-05	0.000554654
TNFRSF1B|7133	0.497349955	0.908120977	1.42003E-05	0.000559482
CYTH4|27128	0.470859777	0.885847088	1.43395E-05	0.000561666
PPP1R13L|10848	3.991904575	1.280067052	1.43319E-05	0.000561666
SLC39A9|55334	1.24453238	1.029490367	1.4316E-05	0.000561666
TLE6|79816	0.4226284	0.778934242	1.43818E-05	0.000562229

PNPLA1|285848	26.34053832	36.02649145	1.44513E-05	0.000563854
IL27|246778	0.3463197	0.459291392	1.46974E-05	0.000572345
C17orf65|339201	0.774061678	0.950981281	1.48669E-05	0.000577828
SAPS1|22870	0.68161105	0.954986816	1.51401E-05	0.000587314
FCN1|2219	0.336312017	0.799186848	1.52395E-05	0.000588901
MPZL3|196264	2.925380672	1.579123803	1.52173E-05	0.000588901
TEX264|51368	0.709225715	0.955468762	1.52783E-05	0.000589267
CTSZ|1522	0.582324858	0.944355122	1.53673E-05	0.000591566
HLA-F|3134	0.530204325	0.922962205	1.55725E-05	0.000597182
RHOV|171177	20.96885659	2.166230146	1.55491E-05	0.000597182
C1orf116|79098	7.856974025	1.537963499	1.56398E-05	0.000598623
RBM42|79171	0.719006735	0.958035088	1.57257E-05	0.000600731
RCOR1|23186	1.302600231	1.040581945	1.57545E-05	0.000600731
CTSS|1520	0.511174347	0.91730883	1.57927E-05	0.000601048
NDUFS7|374291	0.584249419	0.930658992	1.58554E-05	0.000601162
PAQR5|54852	2.54665648	1.225046794	1.58304E-05	0.000601162
C20orf27|54976	0.646143065	0.9421513	1.59553E-05	0.000602041
HSH2D|84941	0.286196771	0.763341152	1.59683E-05	0.000602041
PVRL1|5818	3.586834402	1.199997506	1.5915E-05	0.000602041
SNX16|64089	1.405339473	1.068534386	1.60327E-05	0.00060334
EXT1|2131	1.298975892	1.038518837	1.62077E-05	0.000605013
FZD6|8323	1.699308219	1.081651349	1.62575E-05	0.000605013
IBTK|25998	1.379755779	1.048300651	1.61126E-05	0.000605013
SERPINB13|5275	224.1286072	12.71229129	1.61618E-05	0.000605013
SSFA2|6744	1.716264069	1.069175025	1.6257E-05	0.000605013
TMEM180|79847	0.666608491	0.923243671	1.61791E-05	0.000605013
CYP27A1|1593	0.62737415	0.947117535	1.63877E-05	0.000607613
UBASH3A|53347	0.318741364	0.759113023	1.63754E-05	0.000607613
DLG5|9231	1.557693397	1.068473331	1.65808E-05	0.000613642
RASAL3|64926	0.365169348	0.839630963	1.67904E-05	0.00062026
BRMS1L|84312	1.332867985	1.057163058	1.69637E-05	0.000625514
MFNG|4242	0.436498736	0.862108485	1.70074E-05	0.000625978
CD37|951	0.250570806	0.812243705	1.70982E-05	0.00062817
PCYT2|5833	0.542898765	0.916711274	1.72022E-05	0.000628551
USP38|84640	1.329186681	1.0456582	1.71917E-05	0.000628551
UXT|8409	0.755279262	0.960854923	1.71719E-05	0.000628551
AES|166	0.70750106	0.961858956	1.74694E-05	0.000634725
BTBD7|55727	1.284983397	1.039820351	1.74413E-05	0.000634725
COMMD4|54939	0.699970748	0.953901887	1.74848E-05	0.000634725
MPP1|4354	0.694991099	0.947041431	1.74973E-05	0.000634725
TPRG1|285386	6.236696254	1.900770455	1.7561E-05	0.000635892
MYO1F|4542	0.444321431	0.881414942	1.76233E-05	0.000637001
ALG10B|144245	1.491319386	1.075255263	1.77795E-05	0.000640348
GOPC|57120	1.335643737	1.044925891	1.77742E-05	0.000640348
LOC642587|642587	110.8905081	19.24560456	1.78823E-05	0.000641753
SGMS2|166929	1.832918042	1.130445627	1.78514E-05	0.000641753
RAB8A|4218	0.796441201	0.969716479	1.79616E-05	0.000643454
CRABP2|1382	8.114636176	1.37228332	1.80319E-05	0.000644702
FKBP11|51303	0.611119319	0.935436485	1.80605E-05	0.000644702
C16orf72|29035	1.263621222	1.034923185	1.81151E-05	0.000645506
CYTIP|9595	0.358905891	0.830610241	1.82996E-05	0.00065093
BPTF|2186	1.347523483	1.040873926	1.84173E-05	0.000653962

C6orf108|10591	0.684471011	0.94542293	1.86329E-05	0.000654391
FAM113B|91523	0.422371296	0.851246445	1.86351E-05	0.000654391
GPX4|2879	0.653603786	0.949706584	1.84797E-05	0.000654391
IL1RL2|8808	4.062000407	2.671164638	1.8657E-05	0.000654391
PPOX|5498	0.754826681	0.953981	1.86409E-05	0.000654391
SHKBP1|92799	0.69865105	0.953415009	1.85107E-05	0.000654391
TMEM167A|153339	1.328596923	1.038693075	1.85561E-05	0.000654391
PIK3R5|23533	0.46790168	0.871593208	1.87397E-05	0.000656151
NDRG4|65009	2.949931167	1.225242131	1.8785E-05	0.000656594
DOK2|9046	0.42454878	0.860728736	1.88507E-05	0.000657751
ARRDC2|27106	0.680123401	0.941236213	1.90088E-05	0.000662119
CD38|952	0.356886624	0.816216803	1.91701E-05	0.000666585
DPM3|54344	0.568848907	0.913435684	1.92167E-05	0.000667052
RAET1L|154064	65.84744739	-1.301149931	1.93358E-05	0.00067003
LCTL|197021	3.487862118	1.450634498	1.94384E-05	0.000672429
CD4|920	0.477405512	0.906402635	1.95293E-05	0.000674343
RSPRY1|89970	1.406370178	1.061491611	1.95607E-05	0.000674343
CD86|942	0.53043126	0.892036176	1.96345E-05	0.000675729
HEXIM2|124790	0.657893863	0.927939507	1.97049E-05	0.000675742
LIPG|9388	3.075366595	1.311794808	1.96941E-05	0.000675742
TMEM140|55281	0.657788049	0.942611801	1.97356E-05	0.000675742
C1orf200|644997	0.335366214	0.237827707	1.9788E-05	0.000676387
CCL3|6348	0.428356493	0.855108593	1.99782E-05	0.000676992
CD74|972	0.454570318	0.931146196	2.00075E-05	0.000676992
CYBA|1535	0.525935587	0.921783841	1.99335E-05	0.000676992
PGBD3|267004	1.31957469	1.071416025	1.99056E-05	0.000676992
RNASET2|8635	0.614261978	0.930856547	1.99456E-05	0.000676992
TARP|445347	0.390386602	0.722069902	1.98578E-05	0.000676992
TMEM120A|83862	0.611359586	0.933356169	2.00436E-05	0.000677078
GNG5|2787	0.748717245	0.963327305	2.00866E-05	0.000677393
CD2|914	0.367028307	0.848975351	2.01263E-05	0.000677596
ARHGAP30|257106	0.427615372	0.880729065	2.03227E-05	0.000683067
TMEM101|84336	0.780186455	0.964985694	2.03613E-05	0.000683223

Figure 3A

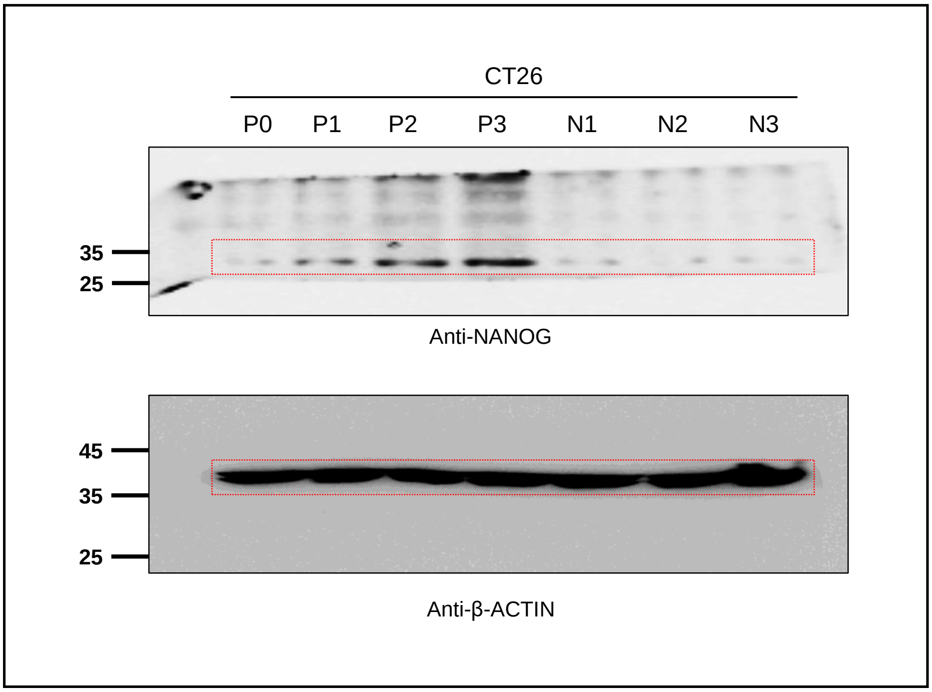

Figure 4D

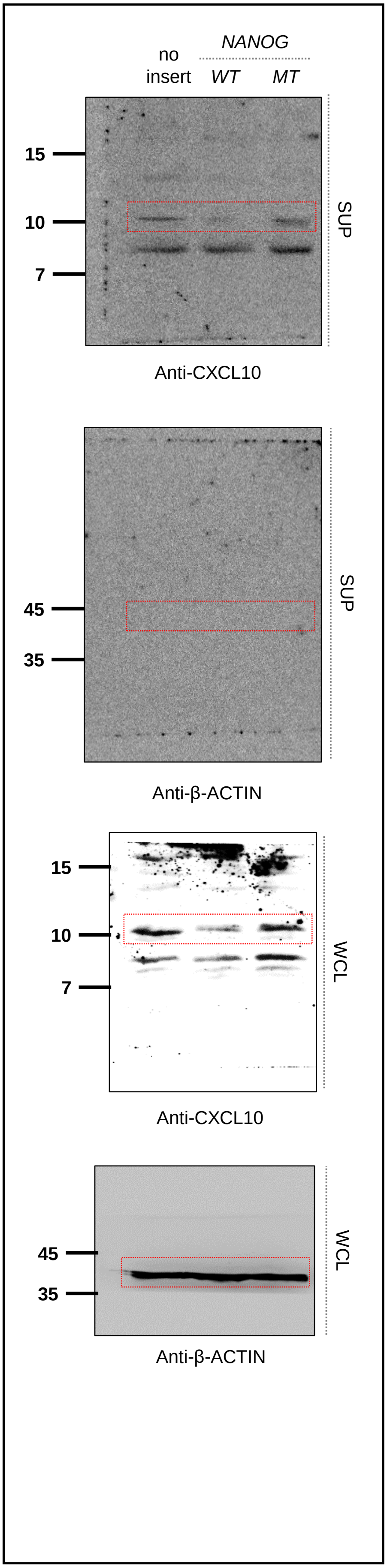

Figure 4E

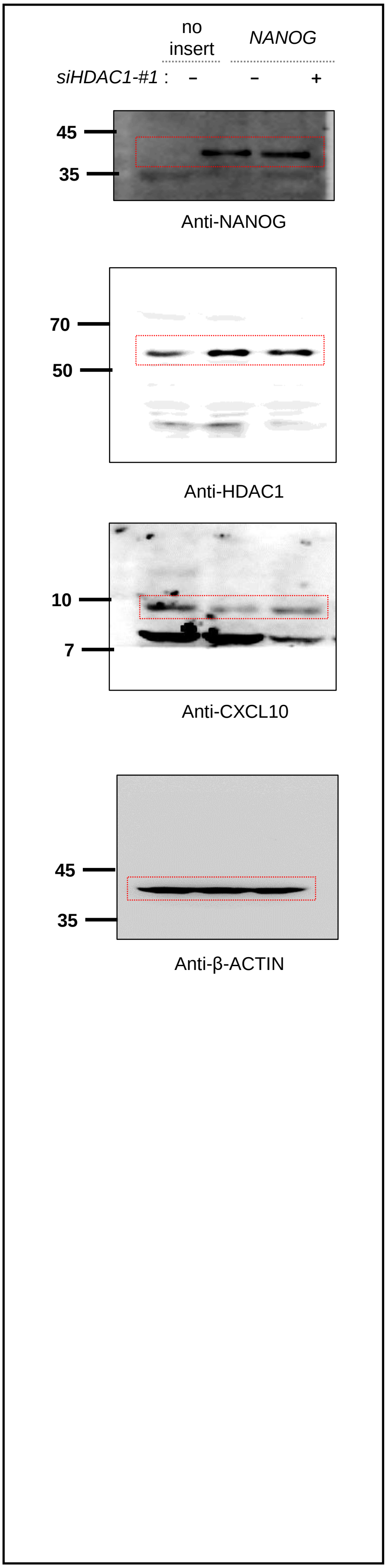

Figure 4I

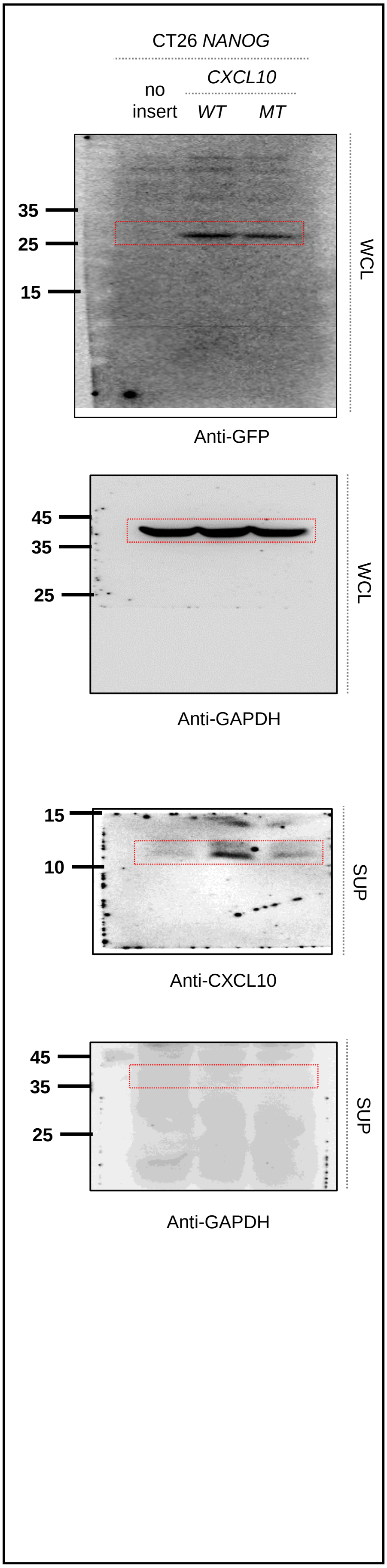

Figure 5A

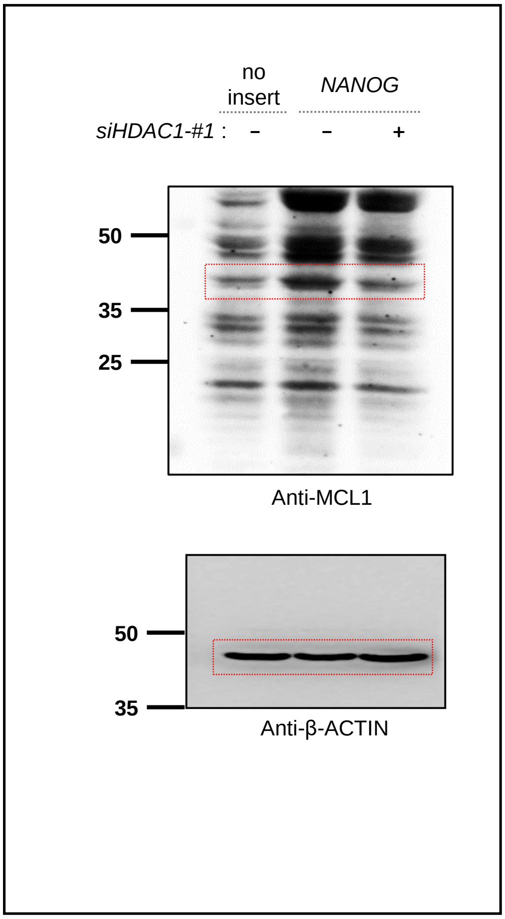

Figure 5C

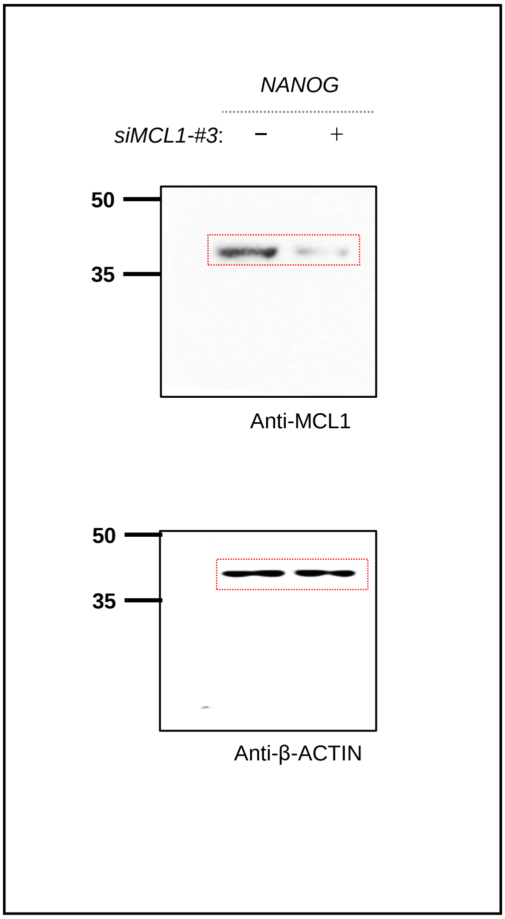

Figure 6A

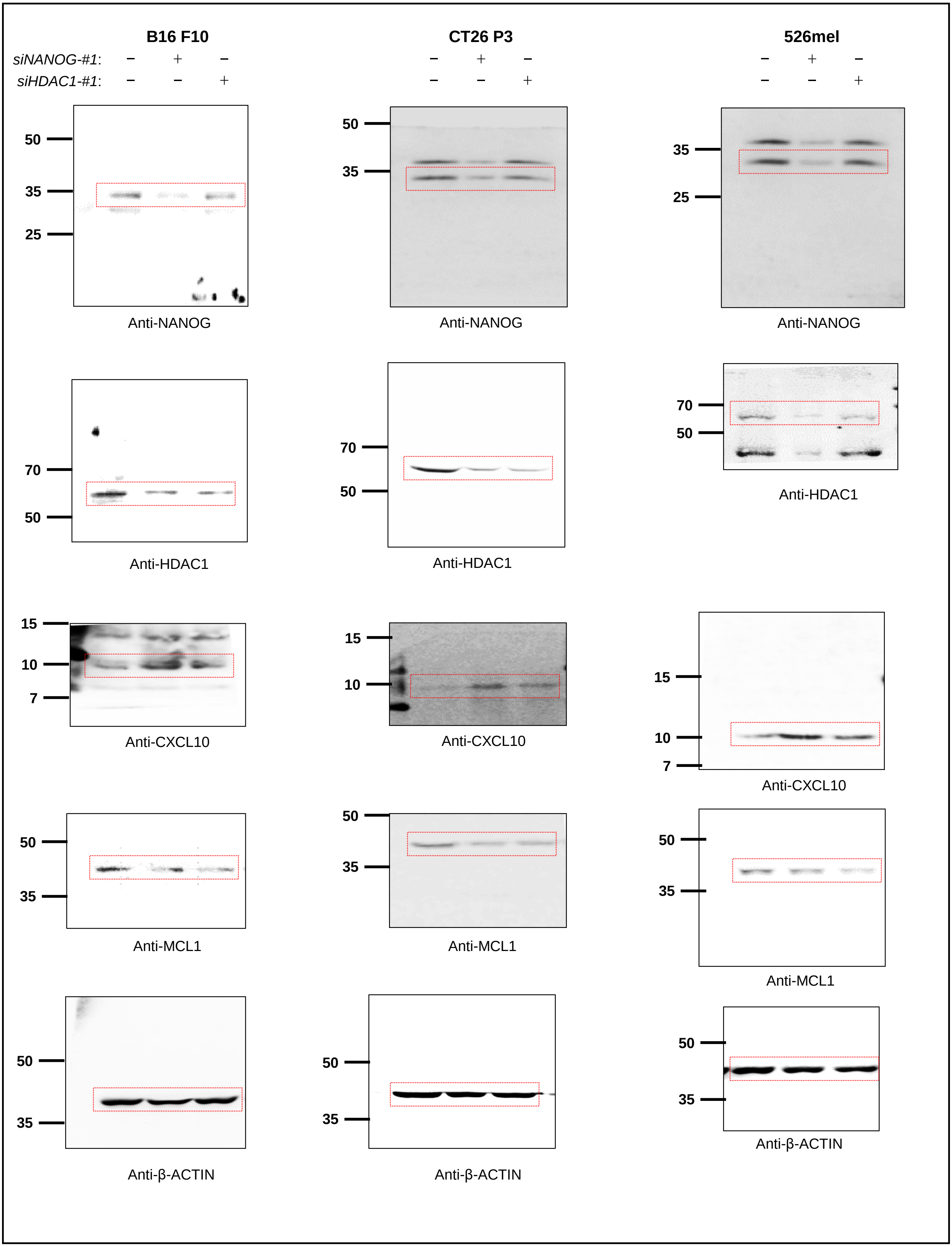

B16 F10
CT26 P3
526mel
siNANOG-#1: − + − − + − − + −
siHDAC1-#1: − − + − − + − − +
50
35
25
Anti-NANOG
70
50
Anti-HDAC1
15
10
7
Anti-CXCL10
50
35
Anti-MCL1
50
35
Anti-β-ACTIN

Figure 6A

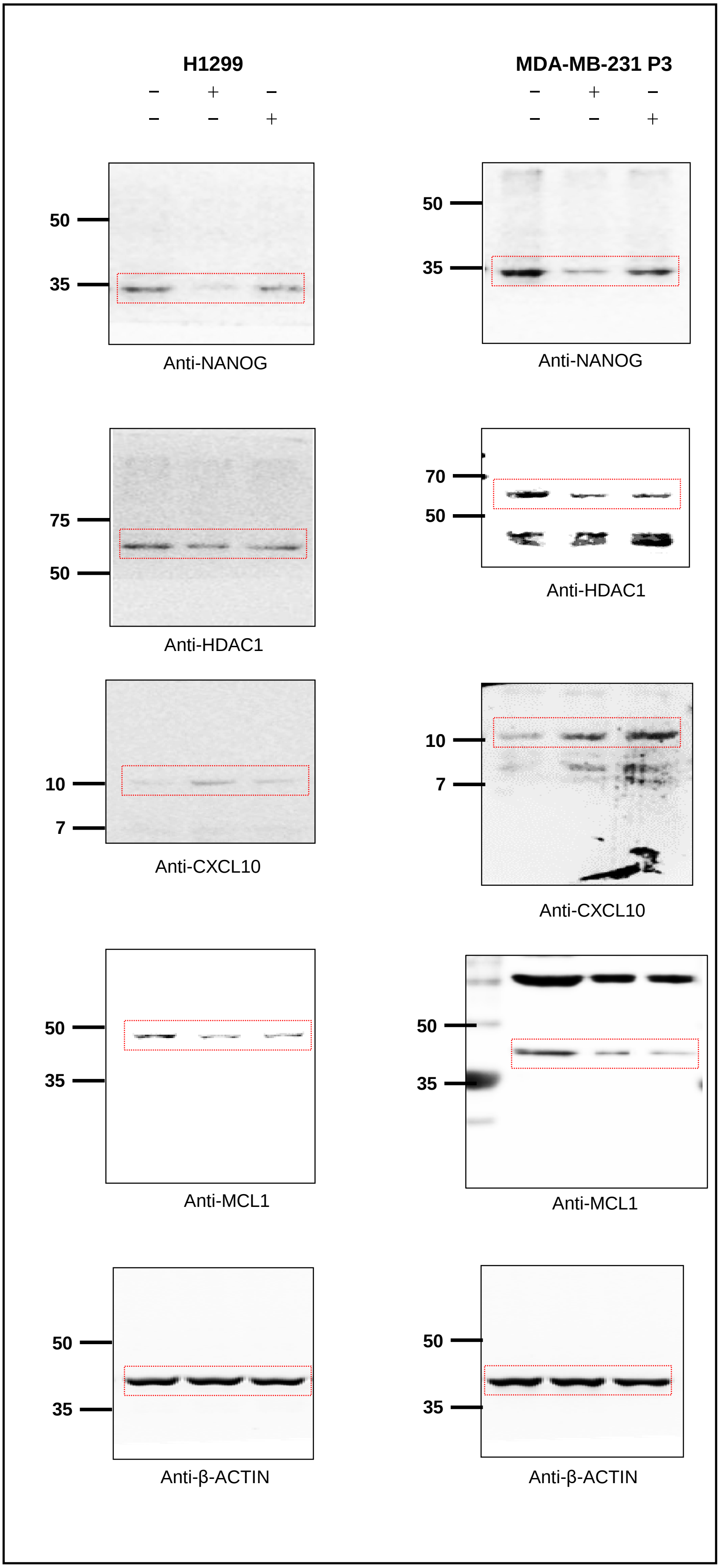

Figure 7B

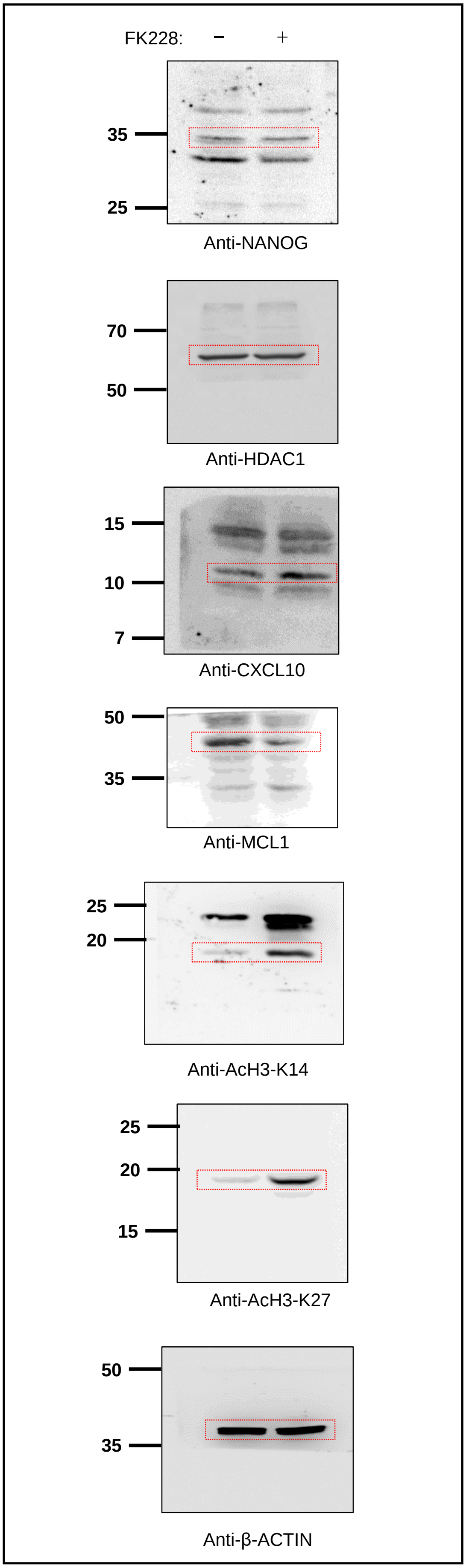

Supplemental Fig 8

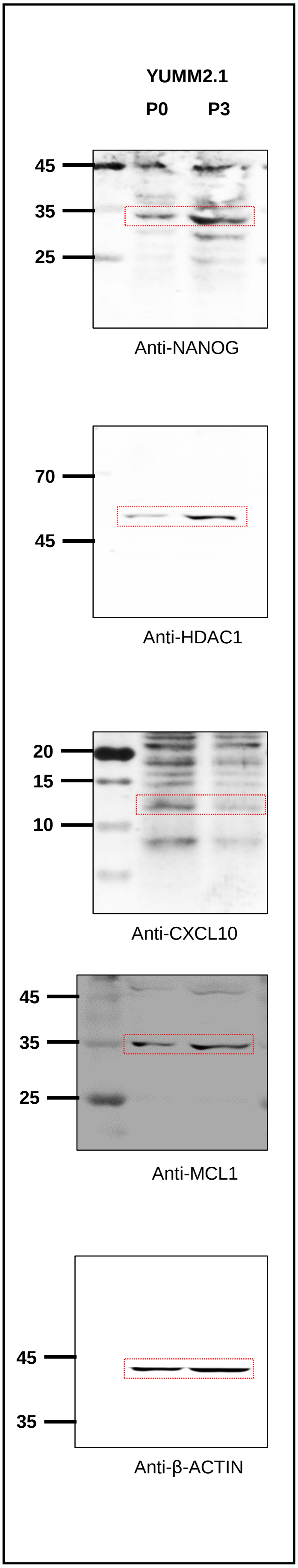

Supplemental Fig 9

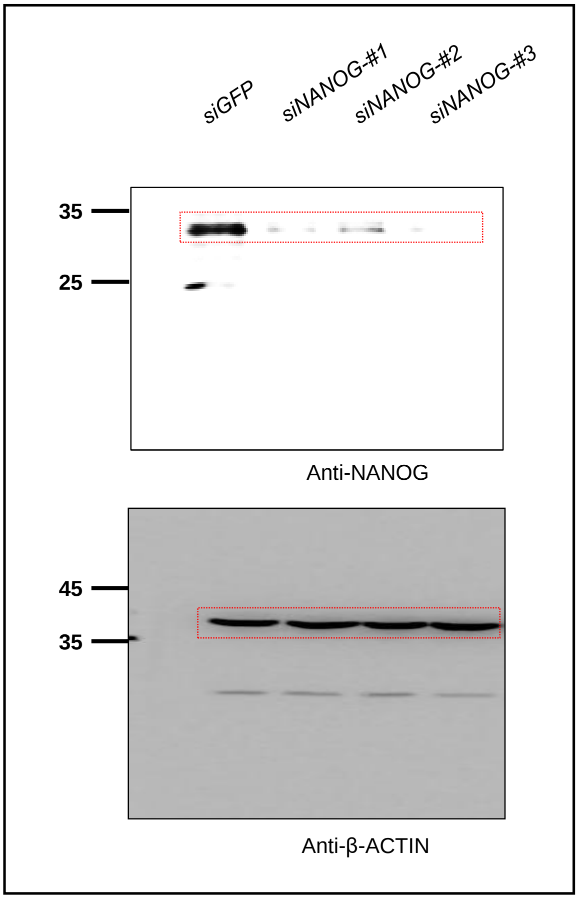

Supplemental Fig 11A

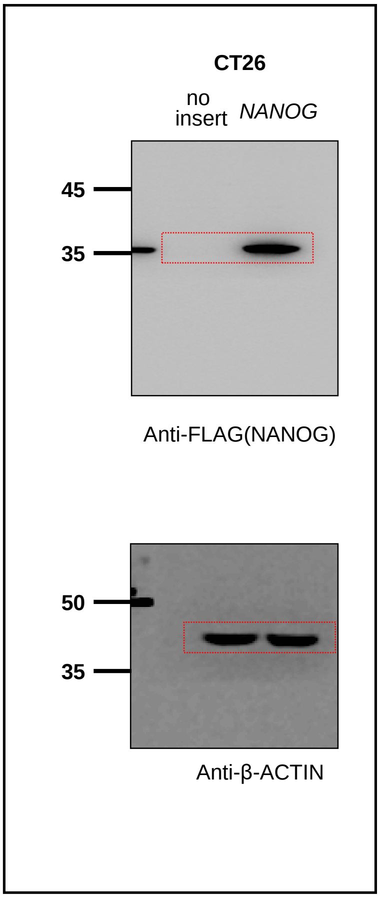

Supplemental Fig 12

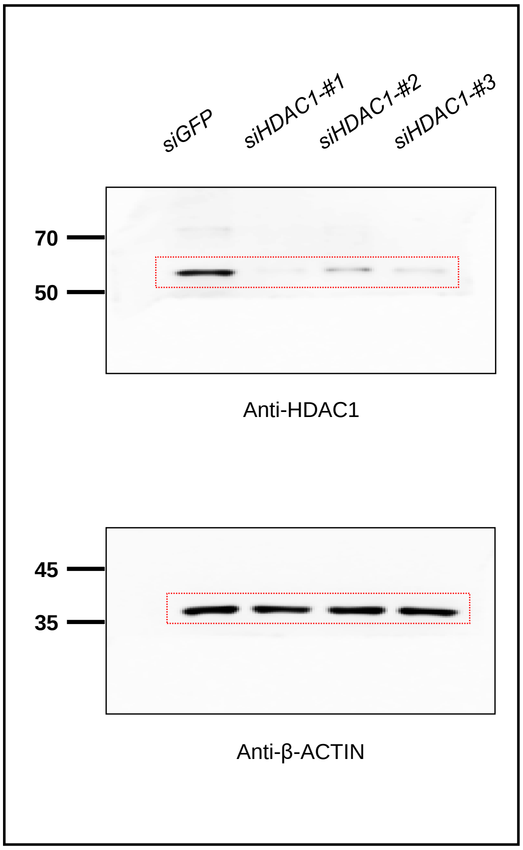

Supplemental Fig 13

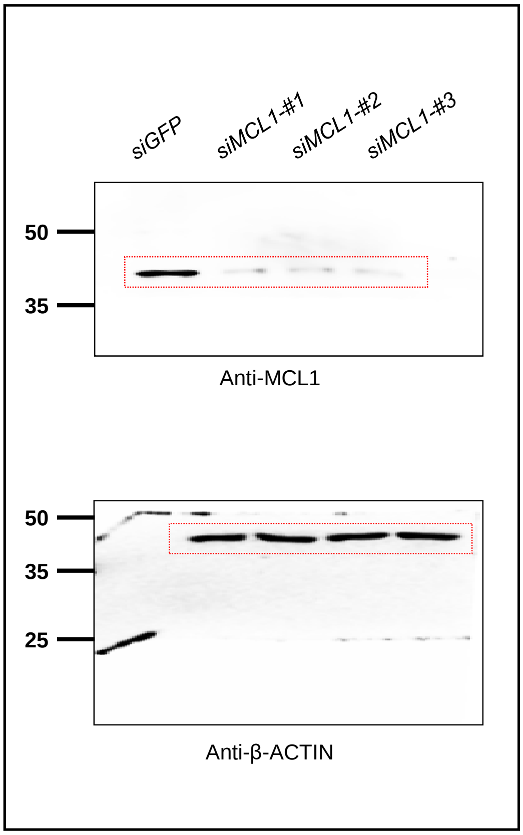

Supplemental Fig 15

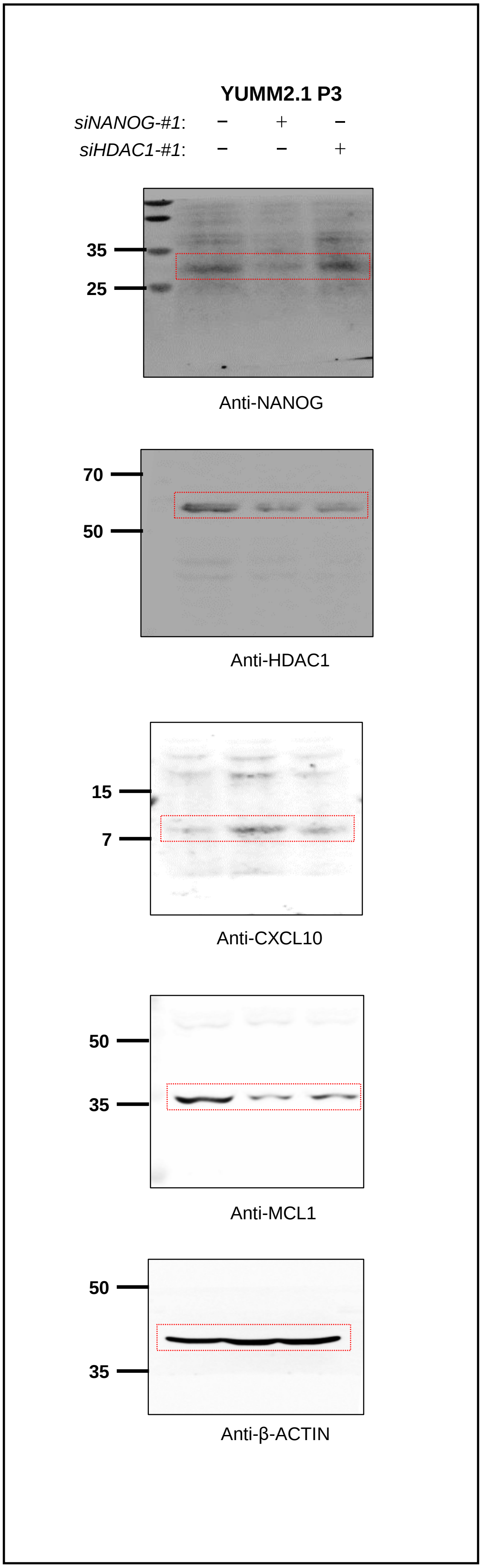

Supplemental Fig 16

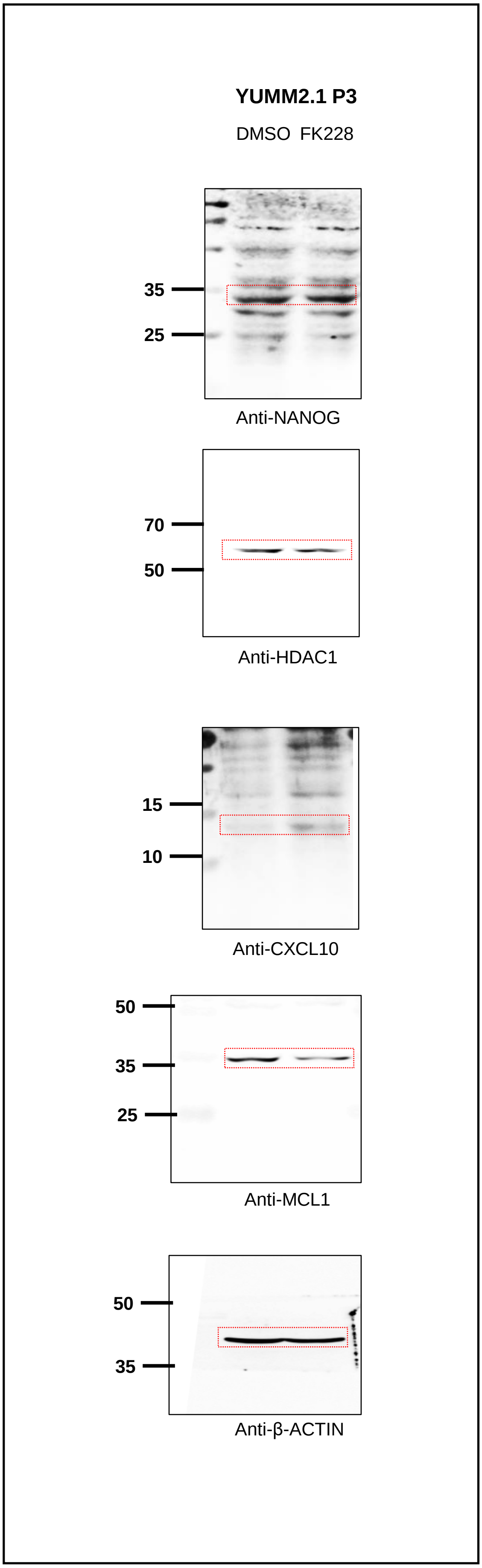

Supplement: Supplemental data [file jci-132-147908-s204.pdf]
